# Metalloenzyme-Inspired Ce-MOF Catalyst for Oxidative Halogenation Reactions

**DOI:** 10.1021/acsami.1c07496

**Published:** 2021-06-28

**Authors:** Sergio Rojas-Buzo, Patricia Concepción, José Luis Olloqui-Sariego, Manuel Moliner, Avelino Corma

**Affiliations:** † Instituto de Tecnología Química, Universitat Politècnica de València—Consejo Superior de Investigaciones Cientificas, Av. de los Naranjos, s/n, 46022 Valencia, Spain; ‡ Departamento de Química Física, Universidad de Sevilla, Profesor García González, 1, 41012 Sevilla, Spain

**Keywords:** Ce-MOF, subnanometric CeO_2−*x*_ clusters, oxidase activity, ligand-to-metal charge transfer, oxidative halogenation

## Abstract

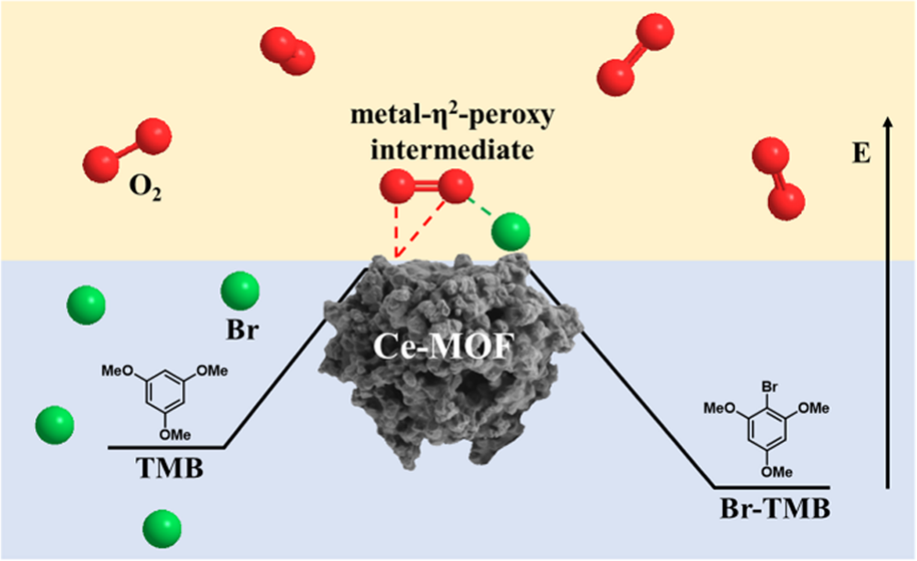

The structure of UiO-66(Ce) is formed by CeO_2–*x*_ defective nanoclusters connected by terephthalate ligands. The initial presence of accessible Ce^3+^ sites in the as-synthesized UiO-66(Ce) has been determined by X-ray photoelectron spectroscopy (XPS) and Fourier transform infrared (FTIR)-CO analyses. Moreover, linear scan voltammetric measurements reveal a reversible Ce^4+^/Ce^3+^ interconversion within the UiO-66(Ce) material, while nanocrystalline ceria shows an irreversible voltammetric response. This suggests that terephthalic acid ligands facilitate charge transfer between subnanometric metallic nodes, explaining the higher oxidase-like activity of UiO-66(Ce) compared to nanoceria for the mild oxidation of organic dyes under aerobic conditions. Based on these results, we propose the use of Ce-based metal–organic frameworks (MOFs) as efficient catalysts for the halogenation of activated arenes, as 1,3,5-trimethoxybenzene (TMB), using oxygen as a green oxidant. Kinetic studies demonstrate that UiO-66(Ce) is at least three times more active than nanoceria under the same reaction conditions. In addition, the UiO-66(Ce) catalyst shows an excellent stability and can be reused after proper washing treatments. Finally, a general mechanism for the oxidative halogenation reaction is proposed when using Ce-MOF as a catalyst, which mimics the mechanistic pathway described for metalloenzymes. The superb control in the generation of subnanometric CeO_2–*x*_ defective clusters connected by adequate organic ligands in MOFs offers exciting opportunities in the design of Ce-based redox catalysts.

## 1 Introduction

Aryl bromides are important intermediates in the synthesis of common drugs, agrochemicals, and organic semiconductors.^1^ In fact, a very large number of bromo and iodoarenes have been isolated from nature (above 1700),^2^ where almost 60 compounds have been approved as clinical drugs.^2^ Moreover, bromoarenes have been employed as versatile reagents for carbon–carbon and carbon–heteroatom bond formation reactions, such as Ullmann, Heck, Stille, and Suzuki.^3^

The preparation of aryl bromides is preferentially carried out following a conventional electrophilic aromatic bromination.^4^ However, this conventional process requires the use of hazardous and toxic molecular bromine (Br_2_). Oxidative halogenation using bromide anions as a bromine source has been described as an interesting alternative.^5^ Sodium periiodate^6^ and lead tetraacetate^7^ have been commonly employed as oxidants in the oxidative bromination of alkenes and alkynes. However, these methodologies require stoichiometric amounts of the oxidant component, also resulting in the consequent generation of large amounts of waste. To overcome this problem, H_2_O_2_- and O_2_-based oxidative bromination reactions have been described as mild and environmentally friendly alternatives.^8^ However, the easy decomposition at elevated temperatures and the high cost of H_2_O_2_ are forcing its replacement by molecular oxygen, the most abundant, cheapest, and green oxidant.^9^

Arene halogenation processes in nature can be achieved with high selectivity under mild reaction conditions using haloperoxidase enzymes.^10^ Nevertheless, the incompatibility with some functional substituents in substrates, high deactivation under severe reaction conditions (pH, solvent, or temperature), and the difficult isolation of the enzyme from the reaction media are some serious limitations for large-scale applications. Inspired by metalloenzymes, vanadium-,^11^ tungsten-,^12^ and molybdenum-biomimetic^13^ catalysts have attracted significant attention. However, these metals show serious health and environmental concerns.^14^ Alternatively, homogeneous copper salts have been reported as active catalysts in the oxidative bromination of arenes using oxygen as an oxidation agent.^15^ Although the selectivities achieved toward monohalogenated products were higher than 90%, the purification steps required to isolate the desired product from the homogenous catalyst enforce the design of novel active heterogeneous transition metal-based catalysts for oxidative halogenation reactions.

Cerium oxide (CeO_2_) is a very interesting metal oxide that combines acid–base and redox properties, where the nonstoichiometric nature of CeO_2–*x*_ generates vacancies and reactive oxygen species.^16−19^ CeO_2_-containing heterogeneous catalysts are widely applied in oxidation processes due to the excellent oxygen mobility and storage capacity of this oxide.^20^ Concretely, ceria nanoparticles are often designed as well-defined nanocatalysts with a focus on maximizing the reactive surface.^21^ Based on these redox-active sites, a nanocrystalline ceria formed by aggregates of primary particles of 8–10 nm has been described as the active catalysts for the oxidative halogenation of activated arenes using bromoalkanes as halogenating agents.^22^

Considering that the relatively large external surface area of the nanocrystalline ceria would offer a higher proportion of redox-active sites compared to bulk CeO_2_ particles, it could be hypothesized that the number of accessible active sites could be considerably increased if the subnanometric CeO_2_ clusters of a few atoms could be stabilized. Having that in mind, if the structure of metal–organic frameworks (MOFs) is analyzed, it can be observed that these materials are composed of very small metallic clusters connected by organic ligands, creating three-dimensional (3D) crystalline microporous materials.^23−27^ Recently, the synthesis of a highly stable Ce-based MOF has been described, UiO-66(Ce), whose structure consists of hexanuclear Ce_6_(μ_3_-O)_4_(μ_3_-OH)_4_ nodes connected by linear 1,4-benzenedicarboxylic acid linkers.^28^ This material shows the existence of ∼10% of Ce^3+^ defect sites, corresponding to ∼50% of Ce_6_ nodes containing at least one Ce^3+^ atom.^29^ This amount of accessible nodes containing a reduced Ce^3+^ ion, and consequently, structural vacancies, may offer unique catalytic properties for diverse redox processes,^30,31^ as for instance for the selective oxidative halogenation of activated arenes.

Herein, we have synthesized a Ce-MOF, UiO-66(Ce), with intrinsic Ce^4+^/Ce^3+^ redox sites as demonstrated by X-ray photoelectron spectroscopy (XPS) and IR-CO analyses. Linear scan voltammetric measurements indicate that Ce^4+^/Ce^3+^ redox conversion is kinetically promoted in UiO-66(Ce), the fact that enhances its oxidase-like activity under aerobic conditions, as demonstrated for the mild oxidation of an organic dye. The presence of terephthalic acid ligands in the MOF structure, with highly delocalized π-electrons, would facilitate this reversible Ce^4+^/Ce^3+^ redox conversion. Nanocrystalline ceria, with nanosized 8–15 nm particles, shows much less oxidase-like activity, in good agreement with its irreversible character of the voltammetric response. Based on the excellent redox properties offered by UiO-66(Ce), its catalytic behavior has been studied for the oxidative halogenation of 1,3,5-trimethoxybenzene (TMB) with 1,3-dibromopropane as a brominating agent using oxygen as a green oxidant. Kinetic studies reveal that UiO-66(Ce) is at least three times more active than nanoceria under the same reaction conditions. For comparative purposes, a Ce_6_-based complex has also been prepared and evaluated for the oxidative halogenation reaction, but non-activity was detected after 21 h underlining the pivotal role of the terephthalate ligands on charge transfer processes as suggested by the electrochemical results. The UiO-66(Ce) material could be reused at least three times, with similar activity in the first two and a slight decrease in the third. The initial catalytic activity could be recovered by a simple Soxhlet extraction. Finally, Raman spectroscopy reveals the formation of metal-η^2^-peroxy species in UiO-66(Ce), which would facilitate the creation of the electrophilic hypohalite species from bromoalkane molecules. These results suggest that the reaction mechanism would follow an analogous pathway to the one proposed for metalloenzymes. The extraordinary control of subnanometric CeO_2–*x*_ clusters within stable MOF-type frameworks, whose electronic properties can be adequately modulated by the presence of the organic linkers, offers unique opportunities for the design of novel redox catalysts.

## 2 Experimental Methods

### 2.1 Synthetic Procedures

#### 2.1.1 Synthesis of Ce-Containing UiO-66 [UiO-66(Ce)]

A solution of Ce(NH_4_)_2_(NO_3_)_6_ (2.44 g, 4.45 mmol) in water (8.43 g) was first prepared. In a 10 mL glass-vessel reactor, terephthalic acid (88.5 mg, 0.53 mmol), dimethylformamide (DMF) (2.82 g), and 1 g of the previously prepared Ce solution were mixed. Ten pyrex reactors were heated at 100 °C for 15 min in a steel block. The resulting pale yellow solid was centrifuged and washed three times with DMF and finally with acetone.

#### 2.1.2 Nanocrystalline Ceria

The nanocrystalline ceria employed in this work was received from Rhodia.

#### 2.1.3 Synthesis of [Ce_6_(μ_3_-O)_4_(μ_3_-OH)_4_ (NH_3_CH_2_COO)_8_(NO_3_)_4_(H_2_O)_6_]Cl_8_·8H_2_O

Following a previous report,^32^ (NH_4_)_2_Ce(NO_3_)_6_ (1.5 g, 2.7 mmol) and glycine (300 mg, 4 mmol) were dissolved in water (0.9 mL). This mixture was diluted with a saturated NaCl solution (10.7 g). Prior to precipitation, the pH was adjusted to ∼0 by the addition of HCl (37%). After 24 h, yellow block crystals were formed and filtered with an excess of ice water. The solid was dried at room temperature under vacuum.

#### 2.1.4 Synthesis of UiO-66(Ce) with Larger Crystallites, Ce-MOF-801 and Ce-MOF-808

The synthesis procedures for these three materials prepared for comparison purposes can be found in the Supporting Information (SI).^33−35^

### 2.2 Characterization

#### 2.2.1 Characterization Techniques

Powder X-ray diffraction (PXRD) measurements were performed using a Panalytical CubiX diffractometer operating at 45 kV and 40 mA and using Cu Kα radiation (λ = 0,1542 nm).

Chemical analyses were carried out in a Varian 715-ES ICP-Optical Emission spectrometer after solid dissolution in H_2_SO_4_/H_2_O_2_ aqueous solution. Elemental analyses were performed by combustion analysis using sulfanilamide as a reference in a Eurovector EA 3000 CHNS analyzer.

The morphology of the samples was studied by field emission scanning electron microscopy (FESEM) using a ZEISS Ultra-55 microscope. The sample was placed on carbon tape stuck on aluminum stubs.

High-resolution transmission electron microscopy (HR-TEM) was performed using a Jeol JEM-2100F operating at 200 kV. The distribution of the particle size for the nanoceria sample was obtained using Software ImageJ. In any case, a minimum number of 100 particles was considered.

The adsorption and desorption curve of N_2_ was measured at 77 K in an ASAP2420 Micromeritics device. The specific surface areas were calculated by the Brunauer–Emmet–Teller (BET) method following Rouquerol’s criterion.

Thermogravimetric and thermal differential analysis (TG–DTG) were conducted in an air stream with a NETZSCH STA 449F3 STA449F3A-1625-M analyzer (Temperature ramp: 25 °C /10.0 (K/min)/800 °C).

Fourier transform infrared (FTIR) spectra were recorded in a PIS 100 spectrometer. The solid samples, mixed with KBr, were pressed into a pellet.

^13^C CP MAS NMR spectra were measured in a 400 MHz Advance III HD spectrometer at 100.62 MHz in a 3 mm probe spinning at 15 kHz. The 90° pulse was 2.3 μs, 2 ms as a contact time and spinal proton decoupling. The number of scans was 3000 with a recycle delay of 3 s. ^13^C chemical shifts were referenced to CHCl_3_. The chemical shifts are reported in ppm.

#### 2.2.2 Cyclic Voltammetry (CV) Study

Linear scan voltammetric measurements were performed with an AUTOLAB PGSTAT 30, from Eco Chemie B.V, in a three-electrode undivided glass cell equipped with a gas inlet and thermostated with a water jacket. The counter and reference electrodes were a Pt bar and an Ag/AgCl/NaCl saturated electrode, respectively. The working electrode was a homemade pyrolytic graphite electrode with a circular geometric area of 0.07 cm^2^. Prior to Ce material coating, graphite electrodes were polished with abrasive P2400 sandpaper, then they were rinsed with Millipore water, and dried. To modify the electrode, a suspension of 5 mg/mL of the solids in a Nafion solution (5 wt % in lower aliphatic alcohols and 15–20% water solution from Sigma Aldrich) was prepared. Then, a 5 μL of this suspension was drop-cast onto the graphite electrode and dried at room temperature for 1 h. The electrochemical properties of the as-prepared Ce composites were investigated by means of cyclic voltammetry (CV) in a solution containing 0.1 M tetrabutylammonium hexafluorophosphate in *N*,*N*-dimethylformamide under an argon atmosphere at 25.0 ± 0.3 °C. The Ohmic drop was compensated using the positive feedback compensation implemented in the instrument.

#### 2.2.3 XPS Measurements

X-ray photoelectron spectra were collected using a SPECS spectrometer with a 150 MCD-9 detector and using a nonmonochromatic Mg Kα (1253.6 eV) X-Ray source. Spectra were recorded using an analyzer pass energy of 30 eV, an X-ray power of 50W, and under an operating pressure of 10^–9^ mbar. During data processing of the XPS spectra, binding energy (BE) values were referenced to the C 1s peak (284.5 eV). Spectra treatment has been performed using CASA software.

#### 2.2.4 FTIR-CO Adsorption Study

IR spectra of the adsorbed CO were recorded at a low temperature (−165 °C) with a Nexus 8700 FTIR spectrometer using a DTGS detector, acquiring at 4 cm^–1^ resolution. An IR cell allowing in situ treatments in controlled atmospheres and temperatures from −165 to 500 °C has been connected to a vacuum system with a gas dosing facility. For IR studies, the samples were pressed into self-supported wafers and treated in a vacuum (10^–5^ mbar) for 1.5 h at 150 °C. After activation, the samples were cooled down to −165 °C under dynamic vacuum conditions, followed by CO dosing at increasing pressure (0.5–2 mbar). IR spectra were recorded after each dosage.

#### 2.2.5 Raman Measurements

Raman spectra were recorded with an “in via” Renishaw spectrometer equipped with an Olympus microscope. The samples were excited by the 514.5 nm line of an Ar+ laser (Spectra-Physics model 171) with a laser power of 2.5 mW. For in situ studies, a Linkam THMS 600 catalytic cell has been used. After sample activation in He (17 mL/min) at room temperature, it has been exposed to an oxygen flow (17 mL/min) at 140 °C. Spectra were acquired at each temperature on different sample spots.

### 2.3 Catalytic Tests

#### 2.3.1 Oxidase Activity of Ce-Containing Materials

Kinetic studies were carried out in a UV container using a suspension of Ce-containing materials (40 μM) in acetate buffer (pH 4.2) at room temperature. 3,3′,5,5′-Tetramethylbenzidine ethanolic solution (1 mM) was then added, and the reaction was monitored at 652 nm in a time scan mode. UV absorption spectra were registered on a Cary 50 spectrophotometer (Varian) using a quartz cuvette of 1 cm optical path and 3 mL capacity.

#### 2.3.2 Oxidative Halogenation Reaction

Reactions with 1,3,5-trimethoxybenzene (TMB) were performed in 2 mL glass-vessel reactors equipped with a magnetic bar, pressure control, and a sample extraction valve. A solution of TMB (126 μmol, 21 mg) in the selected solvent (1.5 mL) was added to each reactor containing the corresponding amount of cerium catalyst (64 and 23 mg for UiO-66(Ce) and nanoceria, respectively). The mixtures were pressurized with O_2_ (6 bar), heated up at 140 °C, and left to stir. Approximately 50 μL aliquots were taken at different times, diluted with ethyl acetate, and centrifuged. The supernatant obtained from batch reactions was analyzed using gas chromatography in an instrument equipped with a 25 m capillary column of 5% phenylmethylsilicone and using biphenyl as an external standard (otherwise indicated).

## 3 Results and Discussion

### 3.1 Synthesis and Characterization of UiO-66(Ce) and Nanocrystalline Ceria

The structure of the crystalline UiO-66(Ce) shows metal clusters based on a hexaoxometallic node (Ce_6_O_8_) connected by terephthalate ligands, presenting pore sizes between 8 and 11 Å (see Figure S1).^28,36,37^ UiO-66(Ce) has been synthesized following a previous description reported in the literature (see the Experimental Section for details).^29^ The resultant solid shows the characteristic PXRD pattern of the UiO-66 material as a pure crystalline phase (see Figure S2). The high microporosity nature of the crystallized material is exposed by N_2_ adsorption characterization (see Figure S3a), where the measured BET surface area, micropore area, and micropore volume are comparable to those previously reported for well-crystallized UiO-66-type materials (∼1117, ∼1070 m^2^/g, and ∼0.53 cm^3^/g, respectively, see Table 1).^28,36,38^

**Table 1:** Physicochemical Properties of the Nanoceria and UiO-66(Ce) Materials

sample	Ce^a^ (% wt)	C^b^ (% wt)	H^b^ (% wt)	N^b^ (% wt)	BET surf. area (m^2^/g)	microp. area (m^2^/g)	microp. vol. (cm^3^/g)
UiO-66(Ce)	27.1	20.4	2.4	0.8	1117	1070	0.53
nanoceria	75.4				98		

a Measured by inductively coupled plasma (ICP) analysis.

b Measured by elemental analysis.

The characterization of UiO-66(Ce) by FTIR spectroscopy clearly shows the disappearance of the ∼1700 cm^–1^ signal assigned to the free carboxylic acid group of terephthalic acid (see Figure S4), indicating the entire interaction of the organic ligand with the cerium clusters. This is consistent with the ^13^C CP MAS NMR spectrum obtained (see Figure S5), where the band assigned to the free carboxylic acid, 172 ppm, is shifted to 170 ppm when terephthalic acid molecules directly interact with the metallic clusters. Moreover, the carbon/metal molar ratio obtained by chemical and elemental analyses (see Table 1) is consistent with the nominal chemical formula of this MOF structure. Finally, the morphology and size of the UiO-66(Ce) particles have been studied by FESEM, observing the formation of ∼200 nm octahedral crystals (see Figure S6).

For comparison purposes, a commercially available nanoceria sample has been selected. The PXRD pattern of this material shows the characteristic peaks of fluorite-type CeO_2_ (see Figure S2). The measured surface area for this nanocrystalline ceria is 98 m^2^/g (see Table 1),^22^ while this material is formed by the aggregation of nanosized particles of 8.8 ± 0.3 nm (see Figures S6 and S7).

**Figure 1:**
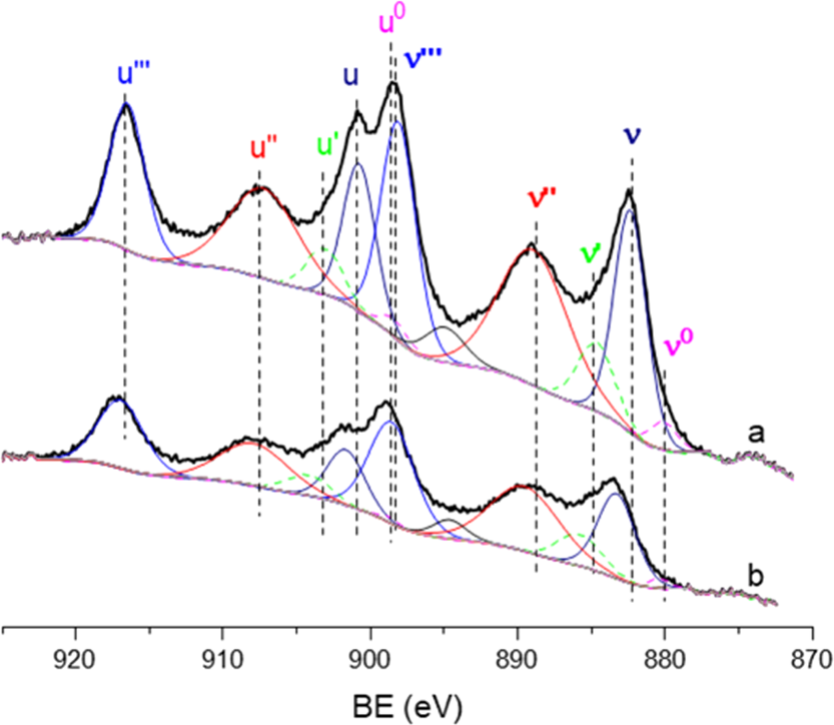
Curve fitting for the Ce 3d XPS spectrum of (a) nanoceria and (b) UiO-66(Ce) samples. Continuous lines correspond to Ce^4+^ and dotted ones to Ce^3+^.

UiO-66(Ce) and nanoceria have been characterized by XPS and IR spectroscopy to deepen into the nature of the Ce sites in both catalysts. The Ce 3d core line in the XPS spectrum of the nanoceria contains six peaks corresponding to the three spin–orbit doublets of Ce^4+^ located at 882.48, 888.9, and 898.1 eV in the Ce 3d_5/2_ component (denoted as *ν*, *ν*″, and *ν*‴) and at 18.5 eV higher BE for the respective Ce 3d_3/2_ component (labeled as *u*, *u*″, and *u*‴) (see Figure 1a).

In addition, two doublets at 880.1 and 884.7eV in the Ce 3d_5/2_ component of Ce^3+^ (denoted as *ν*^0^ and *ν*′) and their respective Ce 3d_3/2_ component at 18.5 eV higher BE (*u*^0^ and *u*′) are observed.^39^ Quantitative analysis results in a Ce^4+^/Ce^3+^ molar ratio of 7.4. Similar spectral features are observed in the UiO-66(Ce) sample with a Ce^4+^/Ce^3+^ molar ratio of 7.9 (see Figure 1b). However, it is worth noting that the BE of the Ce^4+^ and Ce^3+^ components (see Table S1) are shifted ∼1 eV to higher BE in UiO-66(Ce), a fact that can be ascribed to a higher metal site dispersion in its solid matrix.

**Figure 2:**
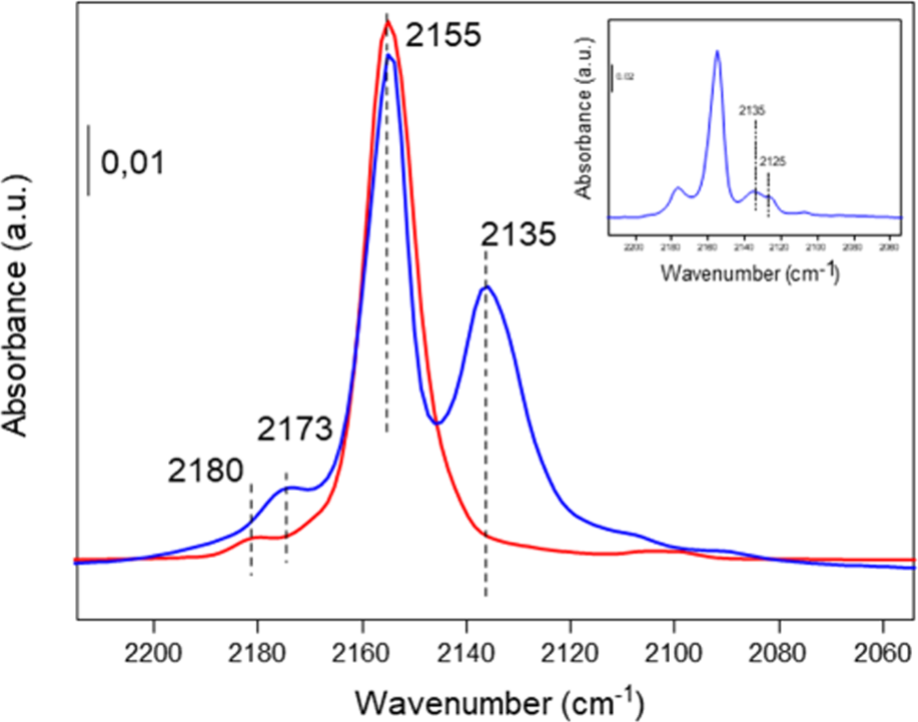
IR spectra of CO adsorption at −165 °C at saturation coverage (2 mbar) on nanoceria (red) and UiO-66 (Ce) (blue). The inset IR spectrum of CO adsorption at 0.5 mbar on the UiO-66 (Ce) sample.

IR spectroscopic studies of CO as a probe molecule have also been performed to better discriminate between the oxidation states of the ceria-based materials and their local environment.^40^ In particular, IR peaks at 2180 and 2155 cm^–1^ are observed in the nanoceria, which have been associated with Ce^4+^ and OH groups, respectively (see Figure 2, red spectrum).

Notably, the IR peak due to Ce^4+^-carbonyl is red shifted toward 2173 cm^–1^ in the UiO-66(Ce) sample (see Figure 2, blue spectrum), related to a lower acidity of the Lewis Ce^4+^ site. The contribution of CO interacting with slight acid OH groups of the UiO-66 material cannot be discarded since a concomitant shift of the OH IR band at 3645 cm^–1^ after CO adsorption is observed. In addition, a component at 2125 cm^–1^ ascribed to Ce^3+^ is detected in the UiO-66(Ce) sample at low CO dosing (see the inset in Figure 2), being overlapped at increasing CO coverage by the 2135 cm^–1^ IR band of physisorbed CO in the MOF channels (see Figure 2, blue spectrum). These results may indicate that while Ce^3+^ species have been detected in the nanoceria by XPS, their absence in the IR-CO study may correspond to their lower surface amount, being preferentially located in the subsurface region of the ceria. Thus, we can infer a higher amount of exposed Ce^3+^ sites in the UiO-66 (Ce) sample than in nanoceria based on XPS and IR studies.

### 3.2 Oxidase Activity of Ce-Containing Materials

**Figure 3:**
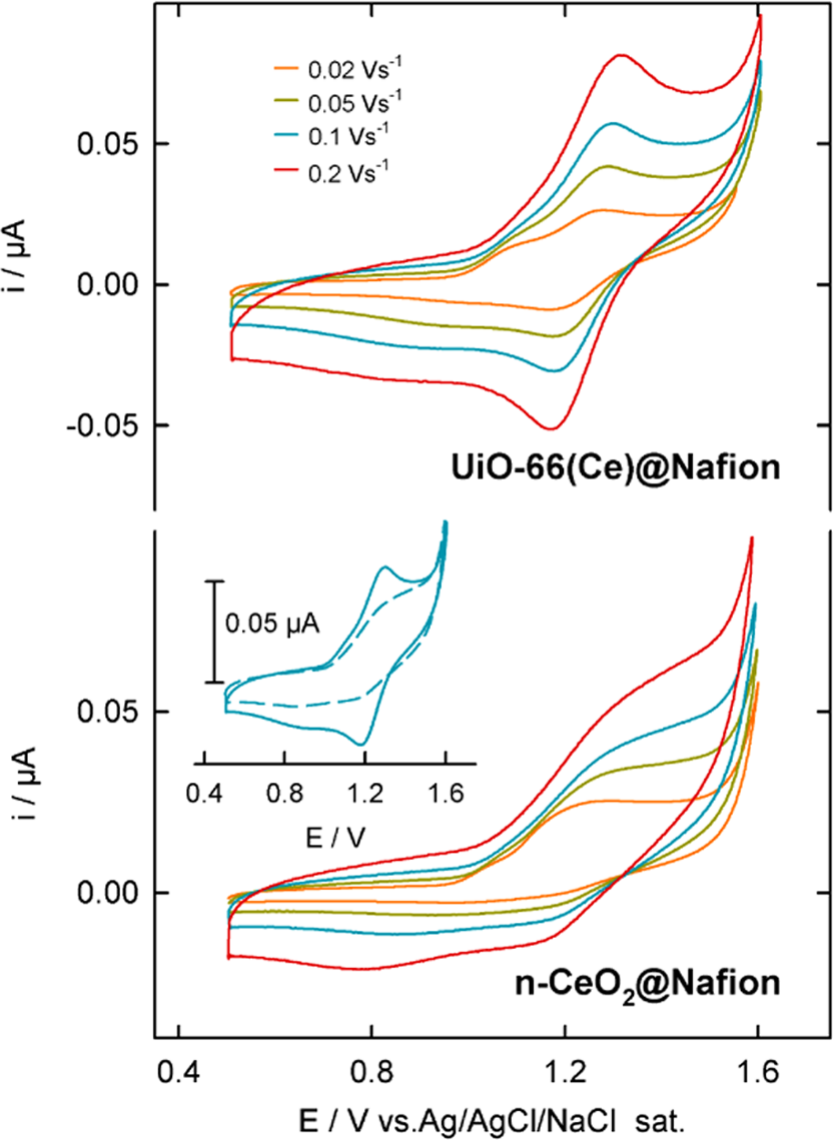
Cyclic voltammograms recorded at the indicated scan rates in a solution containing 0.1 M [Bu_4_N]PF_6_ in *N*,*N*-dimethylformamide at 25 °C of UiO-66(Ce)@Nafion (top panel) or nanoCeO_2_@Nafion composites deposited on a pyrolytic graphite electrode. The inset plot: Comparison of the cyclic voltammograms of both composites recorded at 0.1 V s^–1^.

Since both Ce-based nanomaterials have intrinsic Ce^4+^/Ce^3+^ redox sites, we have investigated the electrochemical behavior of UiO-66(Ce) and nanoceria by cyclic voltammetry, employing a pyrolytic graphite electrode coated with UiO-66(Ce)@Nafion or nanoCeO_2_@Nafion films in a solution containing 0.1 M [Bu_4_N]PF_6_ in dimethylformamide (see Figure 3).

The voltammograms at various potential scan rates of the UiO-66(Ce)@Nafion composite consist of a well-resolved quasi reversible wave with a midpoint potential, an average of the cathodic and anodic peak potentials of 1.22 V (at 0.01 V/s) attributed to the Ce^4+^/Ce^3+^ redox conversion. In contrast, the nanoceria composite displays an irreversible wave with an anodic peak potential of 1.190 V, ∼55 mV more negative to that of UiO-66(Ce), indicating that the latter has a better thermodynamical driving force for the oxidation reaction. The measured open-circuit potentials of 0.53 V for UiO-66(Ce)@Nafion and 0.55 V for nanoCeO_2_@Nafion indicate a similar initial Ce^4+^/Ce^3+^ redox state ratio in both materials, which agrees with the XPS results. Notably, the invariance of voltammetric peak potential separation (Δ*E*_p_) observed for UiO-66(Ce) with a scan rate of up to 0.5 V/s points out to a very fast electron transfer rate across the composite (see Figure 3). This result remarkably contrasts with the irreversible character of the voltammetric response of the nanoCeO_2_@Nafion film, which clearly shows that Ce^4+^/Ce^3+^ redox conversion is kinetically promoted in the synthesized UiO-66(Ce). The better redox performance of the UiO-66(Ce)@Nafion composite can be justified by the decrease of the band gap of the MOF induced by the presence of the terephthalic acid ligand, with highly delocalized π-electrons that facilitates ligand-to-metal charge transfer (LMCT).^41,42^ Moreover, the incorporation of Ce sites into the MOF-nodes promotes LMCT by the low-lying empty 4f orbitals of Ce^4+^. Altogether, these findings reflect that the UiO-66(Ce) material exhibits enhanced redox properties to act as a potential catalyst for oxidation reactions.

**Figure 4:**
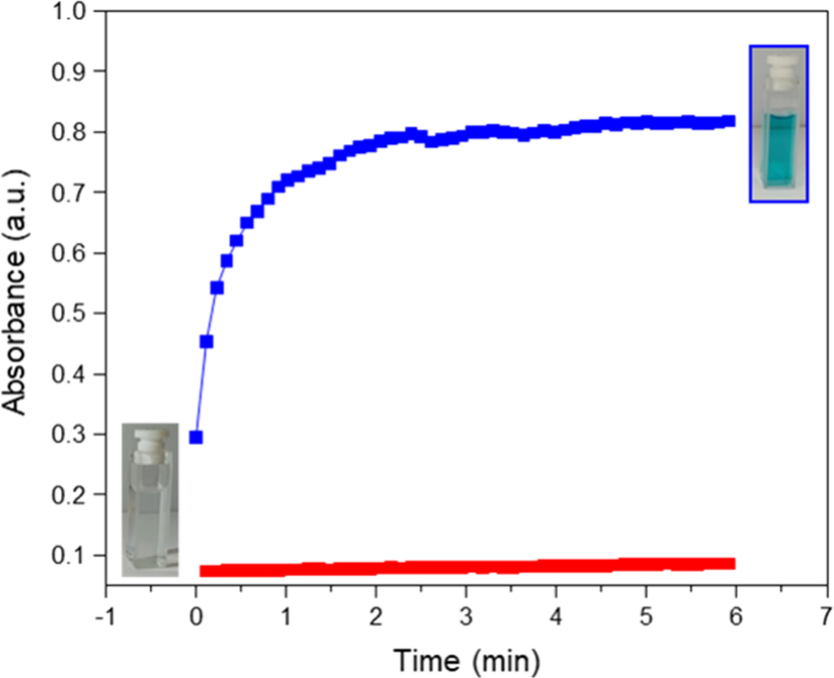
Kinetic profiles for 3,3′,5,5′-tetramethylbenzidine aerobic oxidation employing nanoceria (red squares) and UiO-66(Ce) (blue squares) as nanozymes.

The nanozyme oxidase-like activity of ceria nanoparticles for the fast oxidation of organic dyes without the need for hydrogen peroxide was reported a few years ago, where oxidation was substantially enhanced as nanoparticle sizes decrease from 100 to 5 nm.^43^ Interestingly, the oxidase-like activity of UiO-66(Ce) has been recently employed for colorimetric sensing based on previous nanoceria reported results.^30,44^ Considering the large differences in the voltammetric features between UiO-66(Ce) and nanoceria, we studied the oxidase-like activity of both materials using 3,3′,5,5′-tetramethylbenzidine as the organic dye substrate (see details in the Experimental Section). As seen in Figure 4, UiO-66(Ce) shows an excellent aerobic oxidation of 3,3′,5,5′-tetramethylbenzidine when using 40 μM of Ce in a 1 mM organic dye suspension at room temperature (see Figure S8), whereas nanoceria mostly remains inactive under the same reaction conditions.

These results undoubtedly demonstrate the remarkably higher intrinsic oxidase activity of UiO-66(Ce) compared to the nanocrystalline ceria, in good agreement with voltammetry results. The small hexanuclear ceria nodes in UiO-66(Ce) combined with the presence of organic ligands that can facilitate electronic transfer processes between subnanometric ceria nodes would explain the improved nanozyme oxidase-like behavior of UiO-66(Ce).

### 3.3 Oxidative Halogenation Reaction of 1,3,5-Trimethoxybenzene with UiO-66(Ce) and Nanocrystalline Ceria as Catalysts: Accessible Ce Sites and Role of the Organic Linkers

**Scheme 1:**
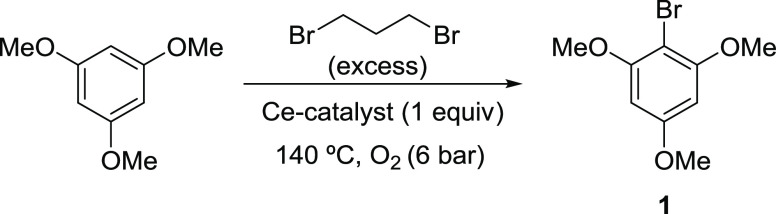
Oxidative Halogenation of TMB using 1,3-Dibromopropane as a Bromination Agent and O_2_ as a Green Oxidant

Taking into account the special redox properties of the Ce-containing nanomaterials, both UiO-66(Ce) and nanocrystalline ceria materials have been tested for the oxidative halogenation reaction of 1,3,5-trimethoxybenzene (TMB) using 1,3-dibromopropane as the brominating agent and solvent at 140 °C under an O_2_ atmosphere (6 bar) (see Scheme 1).

**Figure 5:**
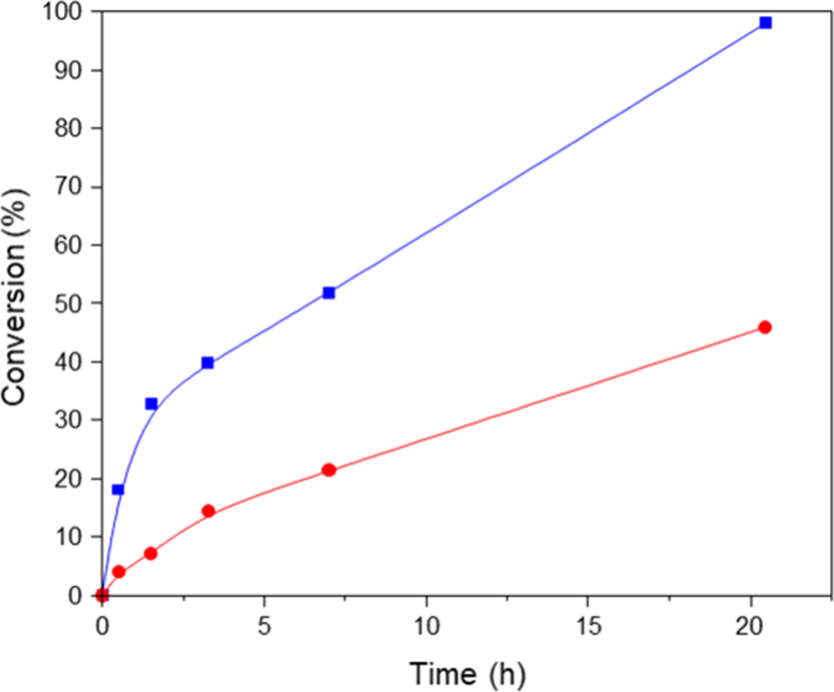
Kinetic profiles for 1,3,5-trimethoxybenzene conversion employing nanoceria (red circles) and UiO-66(Ce) (blue squares) as catalysts. Reaction conditions: TMB (126 μmol) in 1,3-dibromopropane (1.5 mL), fixing a TMB:Ce molar ratio of 1 (64 and 23 mg for UiO-66(Ce) and nanoceria, respectively).

As shown in Figure 5, the TMB conversion is almost completed after 21 h when using UiO-66(Ce) as a catalyst, whereas the nanoceria catalyst only achieves ∼46% TMB conversion at this point. The calculated turnover frequency (TOF) (h^–1^) with UiO-66(Ce) is at least 3 times higher than for nanocrystalline ceria (see Table S2). If product selectivities are analyzed, the only product detected by gas chromatography (GC) and gas chromatography–mass spectrometry (GC–MS) in both cases is the monobrominated TMB (see Figure S9), with 86 and 99% product selectivities after 21 h for UiO-66(Ce) and nanoceria, respectively. Despite the lower selectivity obtained with UiO-66(Ce) compared to nanoceria, the overall product yield after 21 h toward monobrominated TMB is considerably larger for UiO-66(Ce) (84.3 and 45.5% for UiO-66(Ce) and nanoceria, respectively, see Table S2).

To normalize the catalytic efficiency in the oxidative halogenation reaction according to the accessible active Ce sites for both materials, it has been calculated (see Supporting Information for details in Figures S10 and S11) that only ∼7.7% of the overall Ce atoms would be placed on the external surface of the nanoceria particles considering a cubic morphology for CeO_2_ (see Figure S10), whereas a ∼14% of the Ce atoms would be coordinatively unsaturated in UiO-66(Ce) according to the thermogravimetric analysis (TGA) analysis (see Figure S11).^45^ If TOF values are then recalculated considering only the accessible Ce sites, it can be observed that UiO-66(Ce) is still twice more active than nanoceria (see Table S3). This intrinsically higher activity may be related to the presence of terephthalate ligands in the MOF structure, which would facilitate the charge transfer between cerium clusters during the redox process, in good agreement with the electrochemical results.

Since previous voltammetry and normalized catalytic results suggest that the presence of terephthalic acid ligands in UiO-66(Ce) could severely affect its redox properties by LMCT processes, we propose to evaluate the potential catalytic properties of hexanuclear Ce complexes to determine the influence of terephthalate ligands. Thus, a Ce_6_(μ_3_-O)_4_(μ_3_-OH)_4_-based complex with six Ce atoms arranged in a distorted octahedron, similarly to the cluster nodes present in UiO-66(Ce), has been synthesized according to the literature (see the Experimental Section for details).^32^ In fact, this hexanuclear Ce cluster has been previously employed as a metallic precursor in the synthesis of UiO-66(Ce).^46^ The PXRD pattern reveals the formation of the crystalline structure of the hexanuclear Ce complex (see Figure S12).^32^ This Ce_6_(μ_3_-O)_4_(μ_3_-OH)_4_-based complex has been tested as a catalyst for the oxidative halogenation reaction, and after 21 h, no apparent catalytic activity has been observed. The absence of activity would indicate that the LMCT processes facilitated by terephthalate ligands by delocalization of π-electrons could be determinant to undergo the oxidative halogenation reaction.

### 3.4 Catalyst Regeneration, Influence of the Solvent and Other Halogenating Agents Using UiO-66(Ce) as the Catalyst: Preliminary Catalytic Results Using other Ce-MOF Topologies

Considering that non-other by-products are detected by GC, the differences between the product yield and reactant conversion observed when using UiO-66(Ce) could be ascribed to the preferential product adsorption within the pores of the MOF-type catalyst. This hypothesis will also explain the relatively low molar balances observed when working with the MOF. To check the above hypothesis, the used UiO-66(Ce) solid has been recovered after 21 h reaction by filtration and characterized by different techniques. The PXRD pattern of the recovered catalyst reveals that the crystalline nature of the Ce-MOF catalyst is maintained after the reaction (see Figure S13).

However, an increment of the organic content is observed by elemental analysis in the recovered UiO-66(Ce) material after the first recycle (see Table S4), suggesting that some organic substrates are retained within the MOF-type catalyst after the oxidative halogenation reaction. To elucidate the nature of the adsorbed substrates, the recovered solid is also analyzed by ^13^C CP MAS NMR spectroscopy. The band centered at ∼56 ppm in the solid NMR spectrum of the recovered solid can be assigned to the methoxy group in the aromatic ring of 1,3,5-trimethozybenzene (see Figure S14). This characterization unavoidably demonstrates that there is a partial substrate adsorption within UiO-66(Ce). This adsorption process may occur on coordinative unsaturated sites (CUS) of M_6_O_8_ clusters, as it has been recently described for a related methoxy-containing molecule, as 4-methylguaiacol, in Zr-MOF-808,^47^ or by π–π stacking between both organic linkers and substrates through aromatic rings. Taking into account the extra carbon amount adsorbed into the tested catalyst and, assuming that TMB is the substrate adsorbed during the catalytic process, the overall product selectivity value obtained toward monobrominated TMB would be 97% instead of 86% (see Table S5) and the molar balance will be 97%.

**Figure 6:**
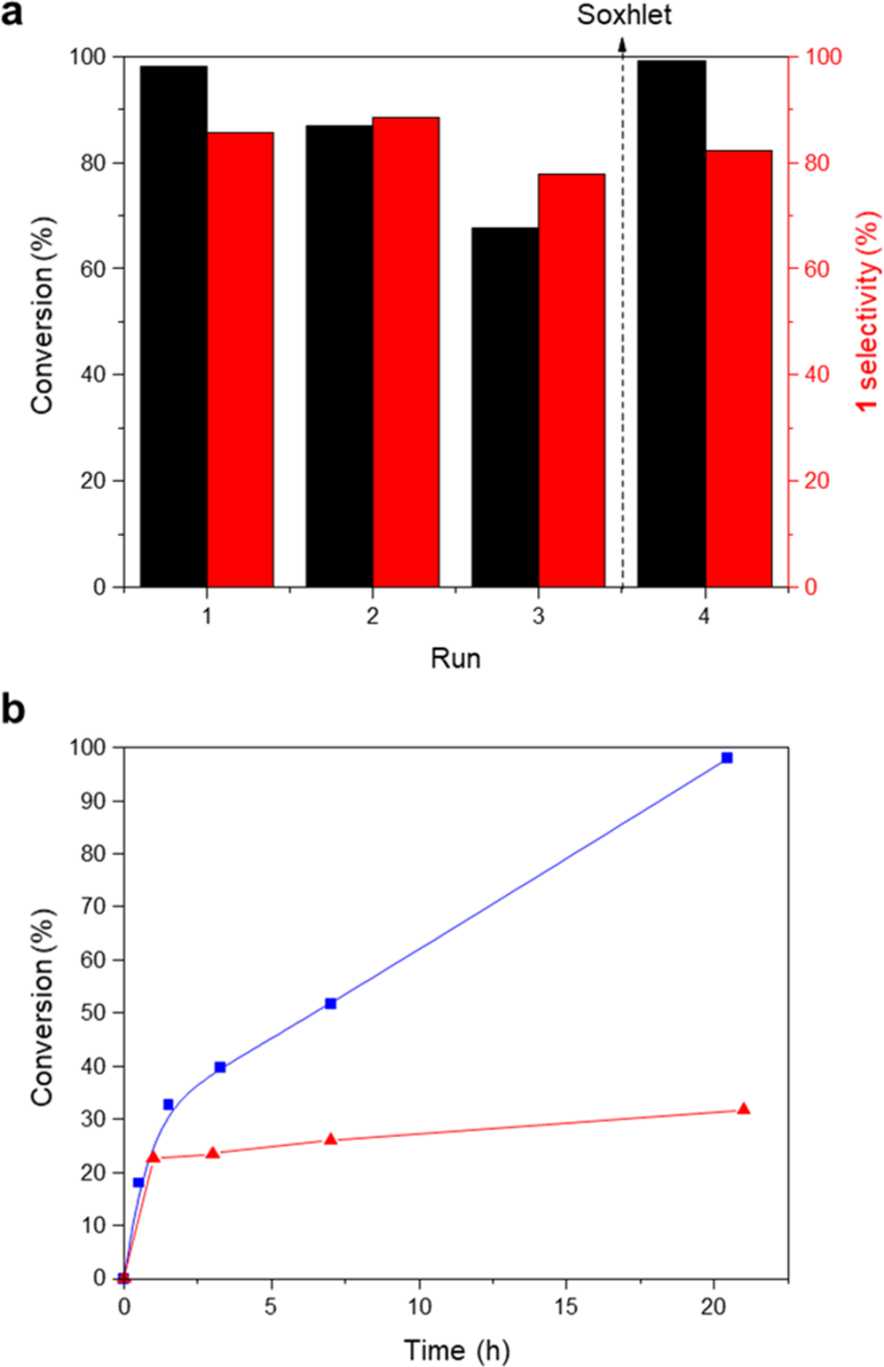
(a) Reusability of UiO-66(Ce). The feeds for the consecutive runs were adjusted to the Ce catalyst. Soxhlet extraction with methanol was performed in the solid catalyst after run 3. (b) Kinetic profiles for 1,3,5-trimethoxybenzene (TMB) conversion employing UiO-66(Ce) as the catalyst (blue line and squares) and after being removed from the reaction mixture after 1 h (red line and triangles). Reaction conditions: TMB (126 μmol) in 1,3-dibromopropane (1.5 mL), fixing a TMB:Ce molar ratio of 1.

The stability of the catalyst for the oxidative halogenation reaction was also probed by reusing the solid with fresh solutions for three consecutive runs. While the conversion and selectivity remained similar in the first and second runs, a decrease of 30% in conversion was observed after the second recycle, along with a drop in the yield of product **1** and in the reaction molar balance (see Figure 6a). As mentioned before, methoxy derivates are being adsorbed during the reaction, partially blocking the catalytic active sites and decreasing the catalytic efficiency of the process. To evaluate if the catalytic activity could be entirely recoverable, washing with methanol of the MOF-type catalyst after the three recycles has been performed to attempt an efficient desorption of the organic compounds from the catalyst.^48^ As seen in Figure 6a, the catalytic activity after the Soxhlet extraction was completely recovered with analogous values to those observed with the fresh catalyst. This recovered material was analyzed by FTIR spectroscopy and TEM/energy-dispersive X-ray spectroscopy (EDX) microscopy. The structural integrity of the recycled catalyst can be claimed based on the similar FTIR spectra and Ce contents observed when comparing fresh and recycled UiO-66(Ce) catalysts (see Figures S15–S17, respectively).

In addition to the reuse tests, a hot filtration test using UiO-66(Ce) material as a catalyst has also been carried out to discard the potential presence of homogeneous catalysis. This catalyst was separated from the reaction mixture after 1 h at 140 °C, allowing the oxybromination reaction to proceed with the filtrate for an additional 20 h. Figure 6b shows that no further reaction occurs after filtration, indicating that there is not an apparent active site leaching and, consequently, UiO-66(Ce) truly behaves as a heterogeneous catalyst. This point has been further confirmed by analyzing the reaction media after catalyst removal, where Ce was not detected by ICP (considering the detection limit of the equipment).

After addressing the heterogeneous catalytic nature of the UiO-66(Ce) material, we have studied the use of different solvents in the reaction media to facilitate the aromatic substrate desorption from the MOF-type catalyst. When 1,3-dibromopropane was replaced by *N*,*N*-dimethylformamide (DMF) as a solvent and only 2 equiv of 1,3-dibromopropane were employed, 98% conversion and 97% monobrominated TMB product selectivity were obtained after 15 h under the same reaction conditions.

In contrast, the conversion obtained after 15 h when using *o*-xylene and *n*-decane as solvents was 28 and 63%, respectively, and the monobrominated TMB product selectivity was only ∼12% for both cases (see Table S6). Since no other by-product was detected by gas chromatography, preferential substrate adsorption into the catalyst is occurring. These results indicate the important role of polar solvents in the desorption process from the active sites for this kind of substrates when using MOF-type catalysts.

The oxybromination reaction with UiO-66(Ce) as a catalyst has also been tested using HBr as a bromination agent. Using 2 equiv of HBr in DMF (1.5 mL), 2-bromo-1,3,5-trimethoxybenzene was obtained in high yields (>99% conversion and selectivity) after 21 h under the same reaction conditions, highlighting that this catalytic methodology can be extended to other bromination agents beyond bromoalkanes.

Finally, and for comparative purposes, UiO-66(Ce) with a remarkably larger crystal size, ∼1 μm,^35^ and other Ce-containing MOFs with different topologies, Ce-MOF-801 and Ce-MOF-808,^33,34^ have been synthesized and tested in the oxidative halogenation of TMB (see synthesis details in the SI and characterization in Figures S18 and S19). The UiO-66(Ce)_1 μm catalyst allows achieving a complete TMB conversion after 21 h, but a considerably lower initial TOF activity is observed for UiO-66(Ce) with a larger particle size (see Tables S2 and S7), suggesting larger diffusional limitations as the UiO-66(Ce) crystal size increases. Additionally, other Ce-containing MOFs with a 6-connectivity (Ce-MOF-808) and 12-connectivity (Ce-MOF-801) on the metal cluster have also been tested for this transformation. On the one hand, Ce-MOF-808, with a lower connectivity in the Ce cluster, showed the highest initial TOF activity and a ∼94% TMB conversion after only 7 h. On the other hand, Ce-MOF-801, with the same cluster connectivity as UiO-66(Ce) but with smaller pore sizes, showed almost complete conversion after 21 h but at least 2 times lower initial TOF activity than UiO-66(Ce) (see Tables S2 and S7). All of these preliminary pieces of evidence suggest that diffusion limitations concerning the particle size, cluster connectivity, and/or pore sizes can play an important role in the final activity for the oxidative halogenation of the Ce-based MOF.

### 3.5 Metalloenzymatic-like Proposed Reaction Mechanism Using UiO-66(Ce) as the Catalyst

**Figure 7:**
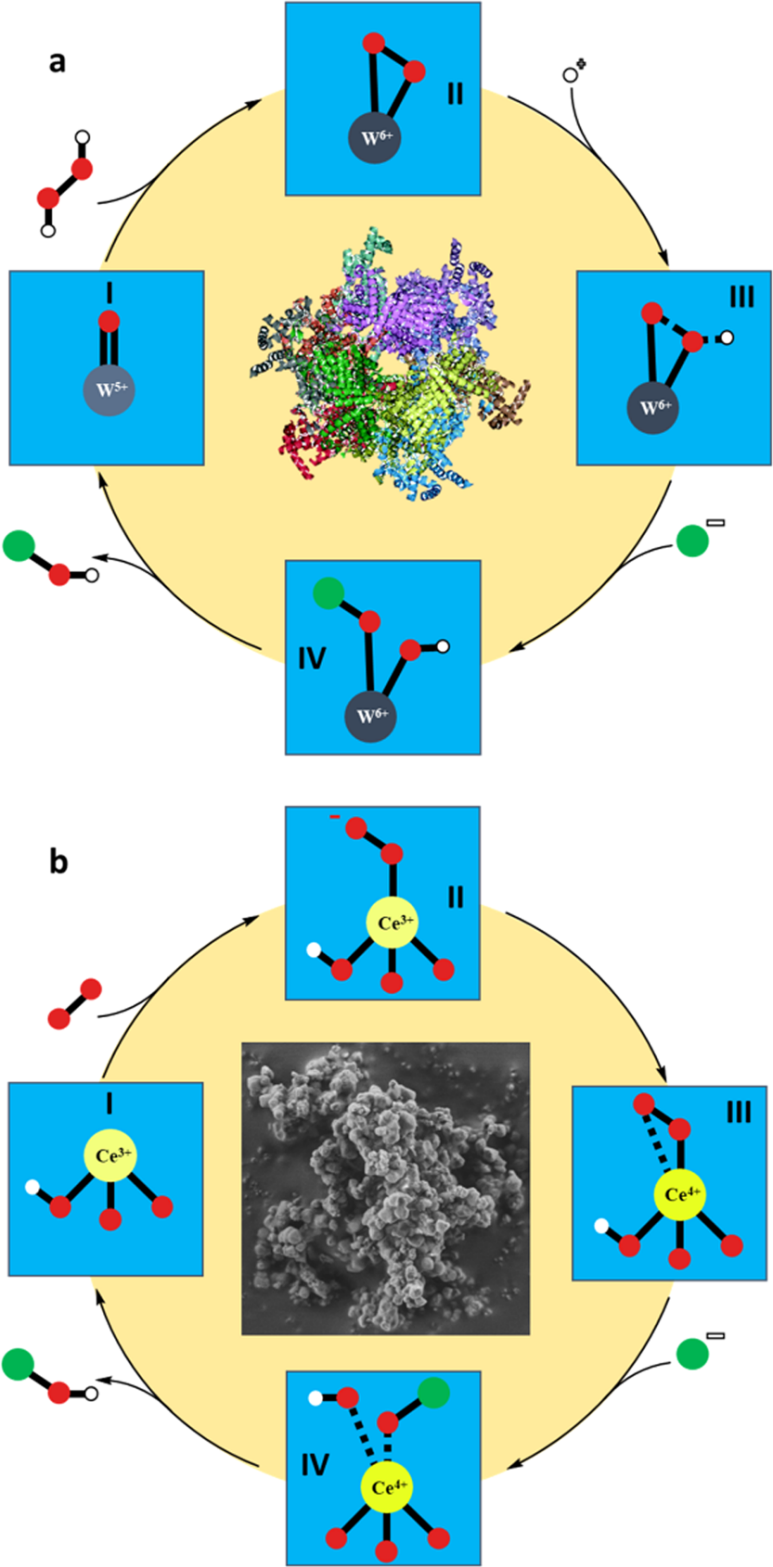
Reaction mechanisms proposed for the generation of hypohalite active species for the halogenation of arenes catalyzed by metalloenzymes (a) and UiO-66(Ce) (b) (Oxygen: red circles, hydrogen: white circles, halide: green circles).

According to the reported mechanism for the halogenation process when using haloperoxidase enzymes, a metal-η^2^-peroxy intermediate is first formed in the presence of hydrogen peroxide with the corresponding metal oxidation (see II in Figure 7a).^12,49,50^ Afterward, this generated peroxide is opened by the halide species, resulting in the formation of an electrophilic hypohalite (see IV in Figure 7a), which acts as an active electrophilic agent.

On the other hand, CeO_2–*x*_ nanorods have demonstrated intrinsic haloperoxidase activity by catalyzing the oxidative halogenation of organic substrates via a reactive hypohalite intermediate. In this case, peroxide species are first formed on Ce^3+^ defective sites when the catalyst was treated with H_2_O_2_.^51^

To better understand the mechanism for the oxidative halogenation of arenes when using oxygen as a green oxidant, Raman studies were performed. Nanocrystalline ceria can provide the peroxide species, more concretely η^2^-peroxy species, when the material was treated with aerobic streams.^16^ Considering this, UiO-66(Ce) and nanocrystalline ceria catalysts have been studied at reaction temperature (140 °C) by Raman spectroscopy under both inert and aerobic atmospheres (see the Experimental Section for details). The signal centered at ∼828 cm^–1^ in the Raman spectrum of the nanocrystalline ceria after being treated in O_2_ at 140 °C can be associated with the presence of η^2^-peroxy species (see Figure S20b).^16^ Interestingly, the appearance of a broad band centered at ∼821 cm^–1^ is also observed when treating UiO-66(Ce) under similar aerobic conditions (see Figure S20d).

This experimental evidence of the formation of η^2^-peroxy species in aerobic conditions in the Ce-based catalysts, together with the excellent oxidase activity, in particular when using UiO-66(Ce) as a catalyst, could indicate that the reaction mechanism may be analogous to that reported for the homogeneous biomimetic catalysts with redox metal active sites.^12^ Indeed, a possible oxidative halogenation mechanism when using oxidase-like UiO-66(Ce) with intrinsic redox sites can be proposed.

First, under aerobic conditions, Ce defective sites on MOF coordinate oxygen to generate peroxide species (see II and III in Figure 7b). This step implies the oxidation of Ce^3+^ to Ce^4+^. The anionic halides from 1,3-dibromopropane interact with one oxygen of the peroxide species at the same time that a proton of the cerium oxoclusters Ce_6_(μ_3_-O)_4_(μ_3_-OH)_4_, with intrinsic OH groups, favors a protonic exchange with the peroxide species (see III and IV in Figure 7b). It is worth noting that the electrophilicity of the Ce-η^2^-peroxy species can be further enhanced by OH sites.^12^ Finally, the peroxide species generated and the halide would yield an electrophilic hypobromite, which would act as an active electrophilic agent to halogenate arenes (see IV in Figure 7b).

## 4 Conclusions

In this work, we have studied the redox properties of a MOF-type material, UiO-66(Ce), containing subnanometric Ce_6_O_8_ clusters connected by terephthalate ligands. XPS analysis shows that UiO-66(Ce) has intrinsic Ce^4+^/Ce^3+^ sites, similar to those observed for nanocrystalline ceria. However, the voltammetric response of UiO-66(Ce) clearly reveals that Ce^4+^/Ce^3+^ redox conversion is kinetically promoted within the MOF-type material, while in the nanoparticulated CeO_2_ the reduction of Ce^4+^ sites is impaired. This fact could be explained because Ce_6_O_8_ clusters connected by terephthalate ligands decrease the band gap of the MOF, facilitating the charge transfer between Ce nodes. According to this, UiO-66(Ce) proves much higher oxidase activity than nanoceria material for the mild oxidation of 3,3′,5,5′-tetramethylbenzidine under aerobic conditions. Based on the excellent redox characteristics of UiO-66(Ce), this material has been studied as a catalyst for the oxidative halogenation of activated arenes using oxygen as a green oxidant. Normalized activities per accessible active sites demonstrate that UiO-66(Ce) shows, at least, a 2-fold catalytic activity increase compared to nanoceria for the oxidative halogenation reaction, indicating that the presence of highly delocalized π-electrons in the MOF ligands would play a pivotal role in this activity enhancement. The Ce-MOF synthesized in this work could be reused in consecutive runs for the oxidative halogenation reaction. The loss of the activity due to the substrate adsorption could be recovered by a simple Soxhlet extraction. Finally, the peroxide species detected by Raman spectroscopy in UiO-66(Ce) under aerobic conditions suggest that the reaction mechanism for the oxidative halogenation reaction when using this Ce-MOF-type catalyst would follow an analogous pathway to the one proposed for metalloenzymes in the oxidative halogenation reaction.

## Supporting Information Available

Different techniques employed to characterize the Ce-based catalysts are included in the Supporting Information, including PXR patterns, N_2_ adsorption–desorption isotherms, FTIR spectra, ^13^C CP/MAS NMR, SEM and TEM images and Raman spectra and the calculations of the accessible Ce sites (PDF)
