# Supplementary material for: Metalloenzyme-Inspired Ce-MOF Catalyst for Oxidative Halogenation Reactions
Source: ACS Appl Mater Interfaces. 2021 Jun 28;13(26):31021–30. doi: 10.1021/acsami.1c07496 (PMC9131423; doi:10.1021/acsami.1c07496)
Supplement: Supplementary file 1 — am1c07496_si_001.pdf [file am1c07496_si_001.pdf]

Supporting Information

Metalloenzyme-Inspired Ce-MOF Catalyst for Oxidative Halogenation Reactions

Sergio Rojas-Buzo,[1] Patricia Concepción,[1] José Luis Olloqui-Sariego,[2] Manuel Moliner,[1]* Avelino Corma[1]*

[1] Instituto de Tecnología Química, Universitat Politècnica de València-Consejo Superior de Investigaciones Científicas, Avenida de los Naranjos s/n, 46022 València, Spain

[2] Departamento de Química Física, Universidad de Sevilla, Profesor García González, 1, 41012 Sevilla, Spain

*Corresponding authors: mmoliner@itq.upv.es, acorma@itq.upv.es

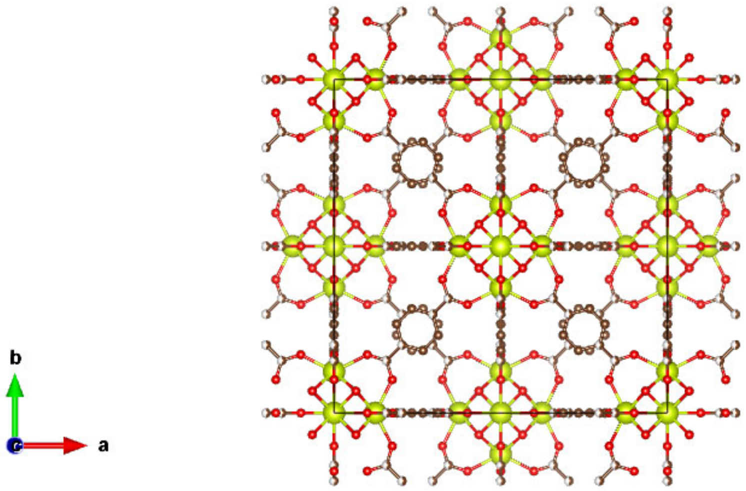

Figure S1. Structure of the UiO-66(Ce) material obtained from the reported crystallographic parameters using the VESTA software.[1]

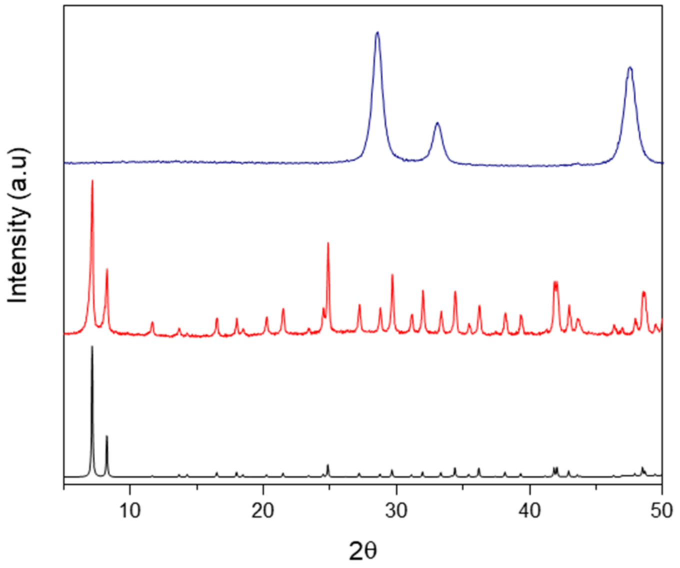

Figure S2. PXRD patterns of the simulated UiO-66(Ce) (black line) and as-synthesized UiO-66(Ce) (red line) and nanoceria (blue line) materials.

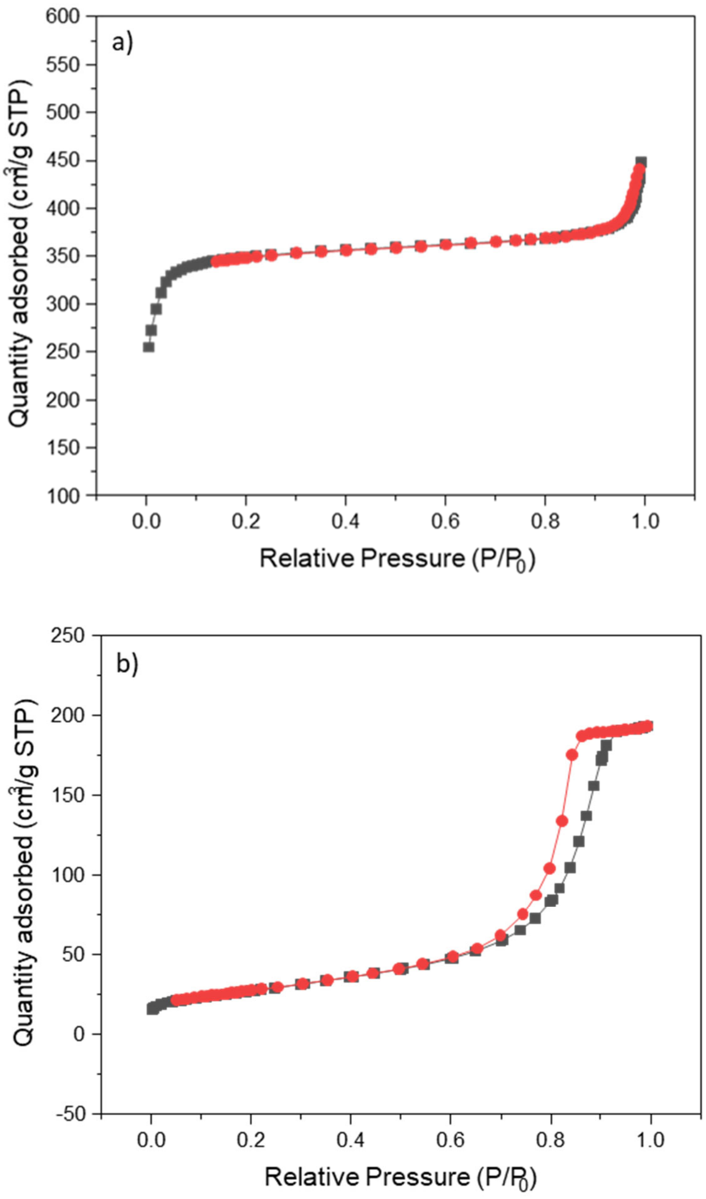

Figure S3. N_2 adsorption (black squares) and desorption (red circles) isotherms of a) UiO-66(Ce) and b) nanoceria materials.

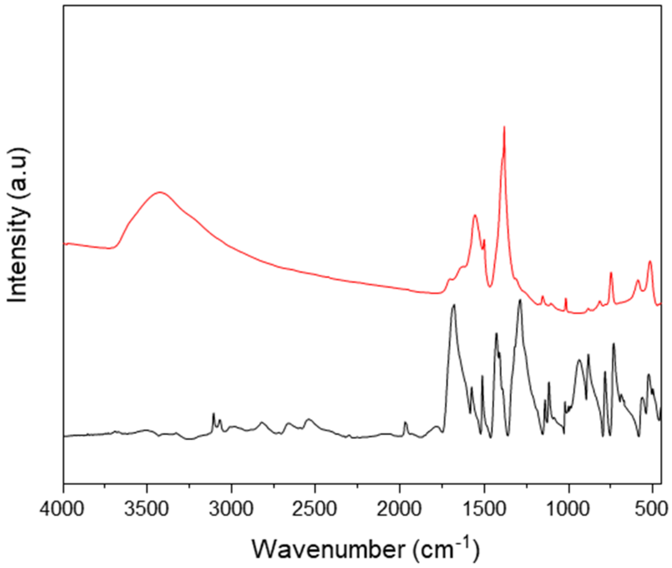

Figure S4. FTIR spectrum of the UiO-66(Ce) material (red line) together with the FTIR spectrum of the organic ligand (black line) employed in its preparation.

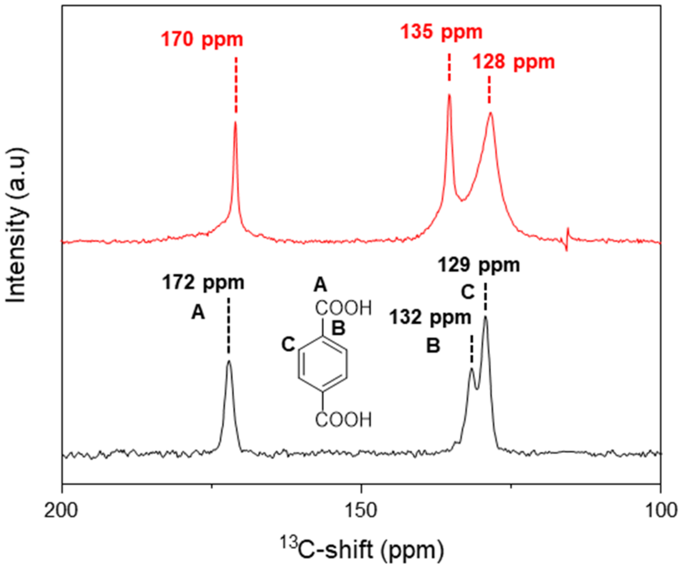

Figure S5. ^{13}C CP MAS NMR spectrum of the UiO-66 (Ce) material (red line) together with the NMR spectrum of the organic ligand (black line) employed in its preparation.

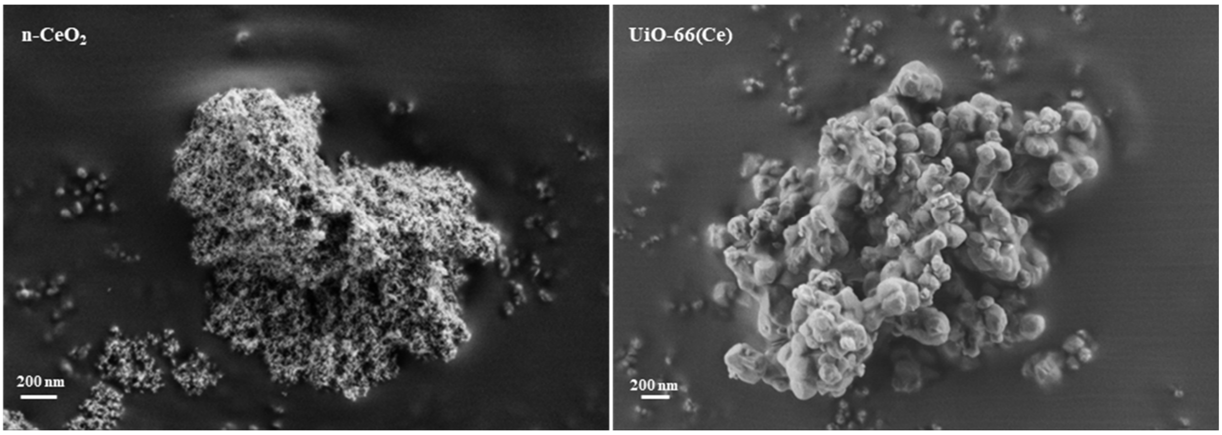

Figure S6. FE-SEM images of the nanoceria and UiO-66(Ce) materials.

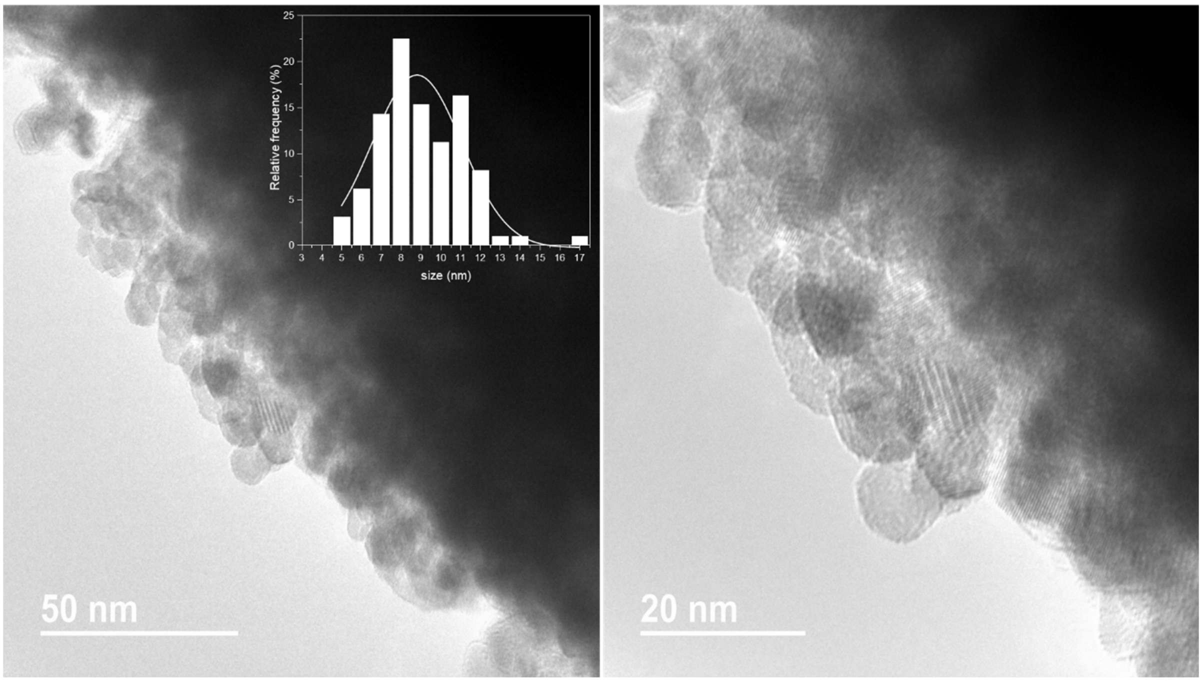

Figure S7. HR-TEM images of the nanoceria material. Particle size distribution fitted by Gaussian function was included.

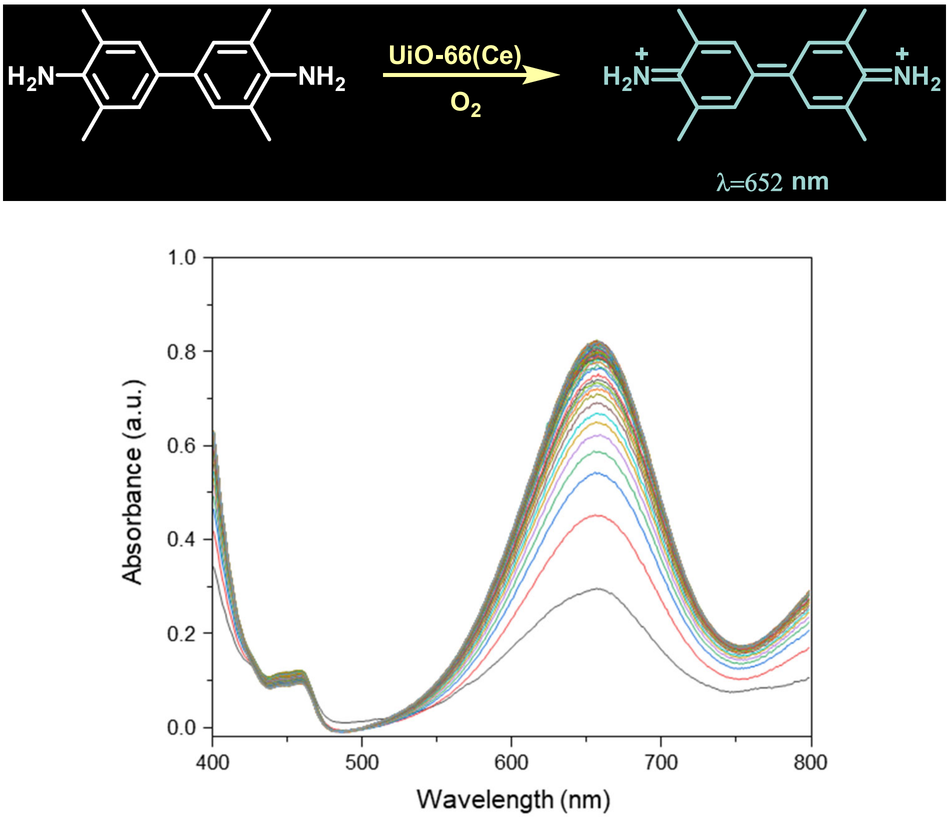

Figure S8. UV-vis absorption spectra of the oxidase form of tetramethylbenzidine recorded at different times when using UiO-66(Ce) as catalyst.

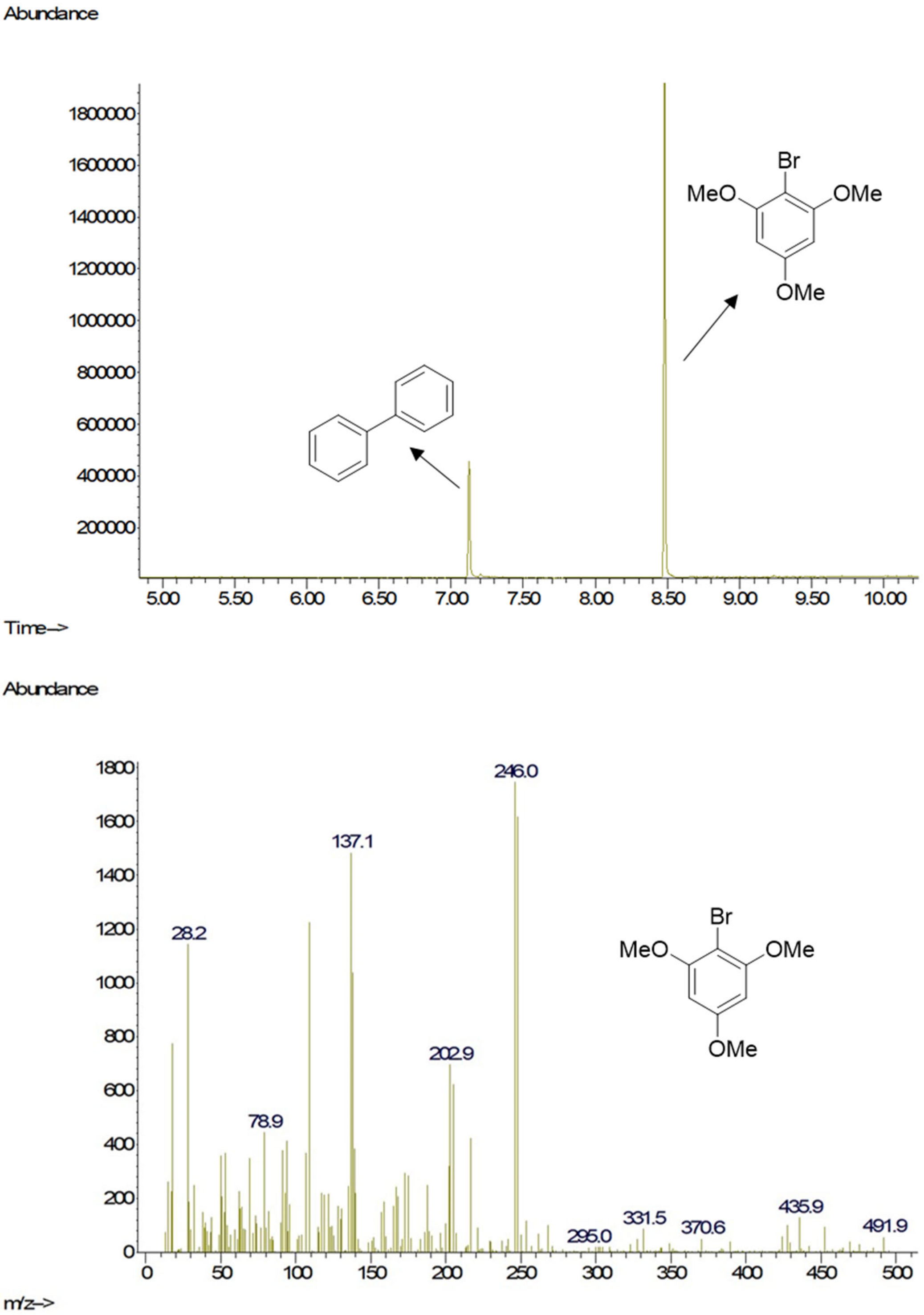

Figure S9. GC and GC-MS spectra of the sample obtained after 21 h in the oxidative halogenation of 1,3,5-trimethoxybenzene when using UiO-66(Ce) as catalyst and biphenyl as external standard.

Calculation of the external surface sites on nanocrystalline ceria

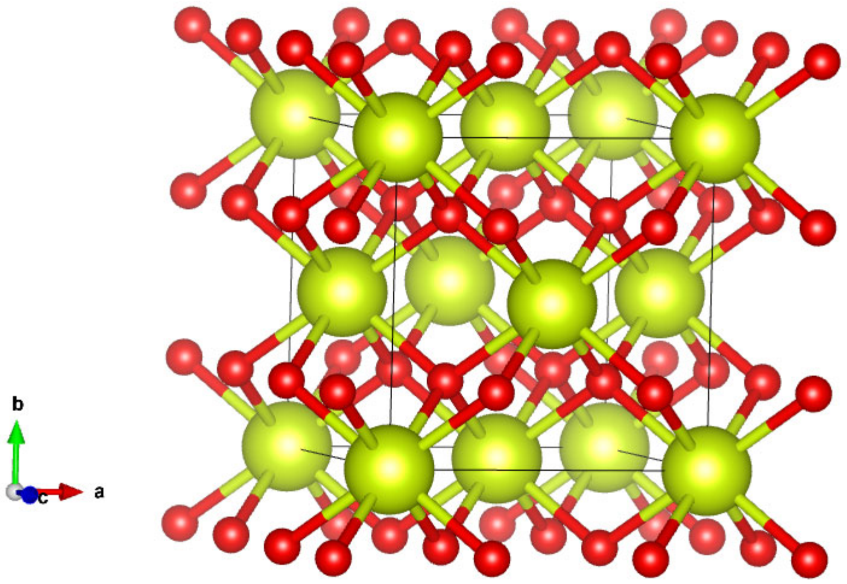

Figure S10. Fluorite-type unit cell assigned to ceria. Image obtained from VESTA software.

Unit cell: Fcc, fluorite type

a, b, c parameters: 3.866 Å

Ce atoms per unit cell: 4

Unit volume: a^3=57.781 $Å^3$

By HRTEM images, it can be extracted that the average size of the nanoceria particles is ~88 Å. Thus, the total volume of a ~88 Å nanoceria particle assuming a cubic morphology would be:

Total volume: a^3=681472 $Å^3$, containing a total of 47176 Ce atoms (assuming 4 Ce atoms per 57,781 $Å^3$).

Considering 111 facet in an optimized unit cell where there are 9 Ce atoms per 114.104 $Å^2$, the density of superficial Ce atoms is:

$$\sigma_{sup} = \frac{9\ Ce\ atoms}{114.104\ Å^2} = 0.0789\ Ce\ atoms/Å^2$$

Moreover, the density per volume in the cubic unit cell will be:

$$\rho_V = \frac{4\ Ce\ atoms}{57.781\ \text{Å}^3} = 0.0692\ Ce\ atoms/\text{Å}^3$$

So, the ratio of Ce atoms in the external surface versus the bulk particles has been calculated assuming a cubic morphology:

$$\frac{Particle\ surface}{Particle\ volume} = \frac{6 * a^2 * \sigma_{sup}}{a^3 * \rho_V} = \frac{6 * \sigma_{sup}}{a * \rho_V} = \frac{6 * 0.0789}{88 * 0.0692} = 0.0777$$

That indicates only 7.77% Ce atoms are in the cerium oxide surface (3665 Ce atoms).

Calculation of the accessible sites on UiO-66(Ce)

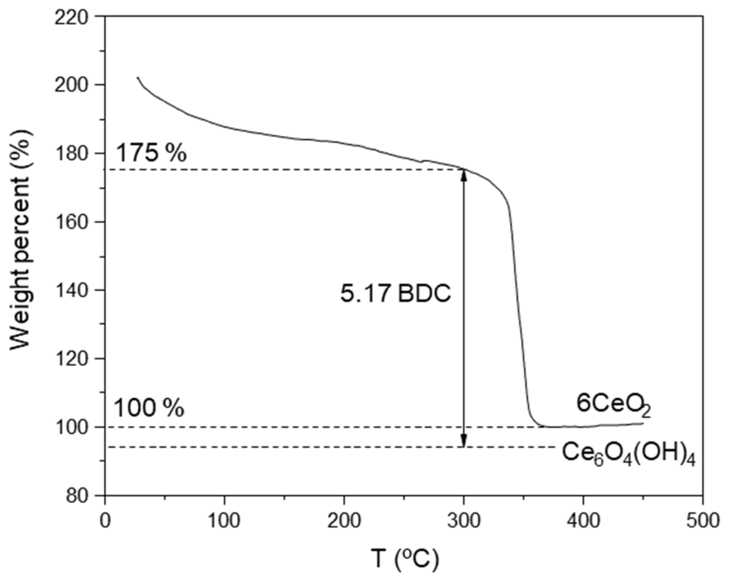

Figure S11. Thermogravimetric analysis of the UiO-66(Ce) as-synthesized.

Linker defects were calculated following previous reports.[2,3] 100 g of CeO_2 (0.58 mol) correspond to 0.097 mol of Ce_6 hexanuclear clusters. For that normalized amount, the corresponding mass of $Ce_6O_4(OH)_4$ would be 94 g. Therefore, for each 100% left after heating up to 400°C, an extra of 6% is attributed to the organic linker. The following formula is used to calculate the amount of linker (normalized to 100 g of CeO_2 residue):

$$La = \frac{TGA - 100 + 6}{MW} = \frac{175 - 100 + 6}{164} = 0.49$$

where La is the amount in mol of linker detected (normalized to 100 g of CeO_2 residue), TGA is the weight loss of the linker determined in Figure S5 (normalized to 100 g of CeO_2 residue), number 6 is the mass difference between CeO_2 and $Ce_6O_4(OH)_4$ (normalized to 100 g of CeO_2 residue) and MW is the molecular weight of the linker (164 for BDC^{-2}).

The following formula is used to calculate the number of linkers per Ce_6 node:

$$NL = \frac{Nt * La}{Lt} = \frac{6 * 0.50}{0.58} = 5.17$$

where, NL is the number of linkers per Ce_6 node, Nt is the theoretical number of linkers per Ce_6 node (this should be 6 for BDC^{-2}), La is the amount in mol of linker detected (normalized to 100 g of CeO_2 residue)

and Lt is the theoretical amount in mol of linker for the normalized weight of 100 g of CeO_2 residue (this would be 0.58 mol for BDC^{-2}).

According to these calculations, it can be concluded that 14% of the overall Ce sites are coordinatively unsatured (a total of 5.17 organic linkers are present in the UiO-66(Ce) structure instead of the theoretical 6 organic linkers of an ideal $Ce_6O_4(OH)_4(BDC)_6$ structure).

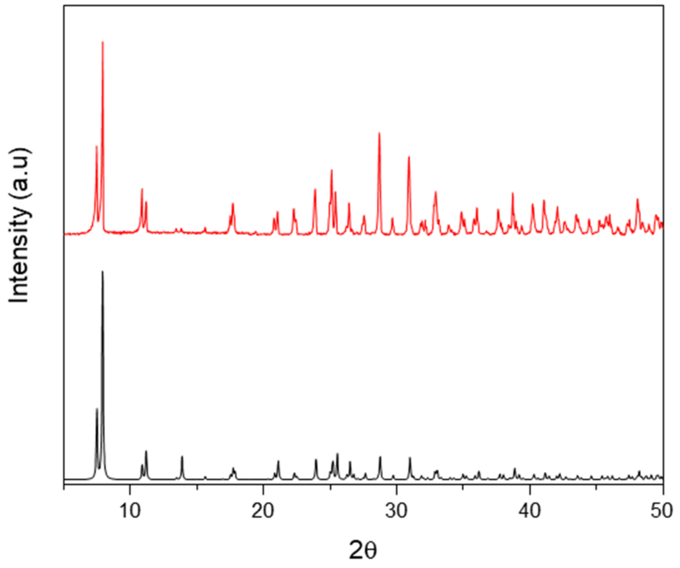

Figure S12. PXRD pattern of the simulated (black line) and as-synthesized (red line) hexanuclear Ce complex synthesized according to the description reported in Estes et al.[4]

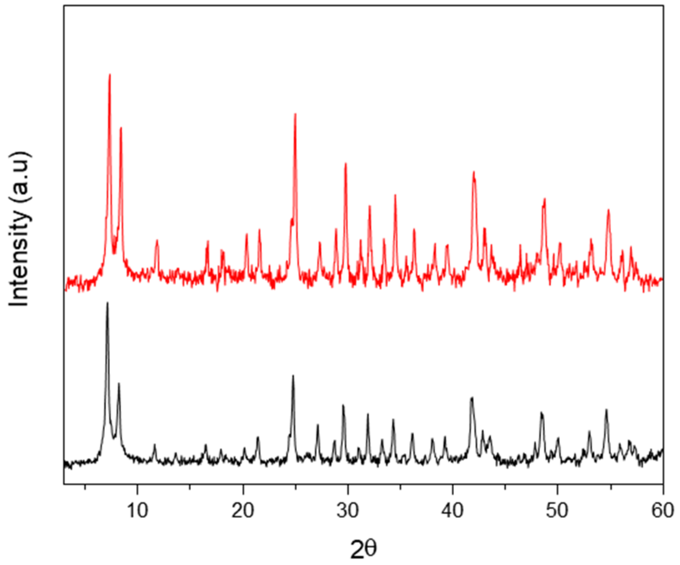

Figure S13. PXRD pattern of the as-synthesized UiO-66(Ce) (black line) and after the oxidative halogenation reaction (red line).

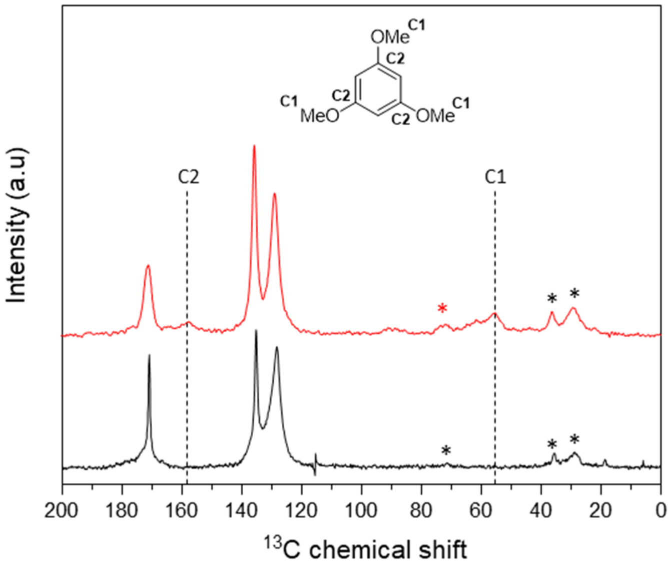

Figure S14. ^{13}C CP/MAS NMR spectra of the as-synthesized UiO-66(Ce) (black line) and after the oxidative halogenation reaction (red line). Asterisks represent spinning bands.

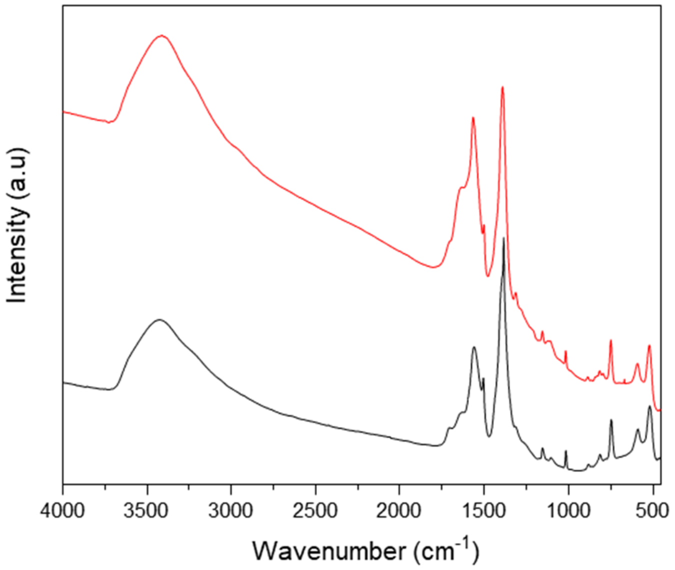

Figure S15. FTIR spectrum of the fresh UiO-66(Ce) material (black line) together with the FTIR spectrum of the recovered MOF after 4 consecutive runs (red line).

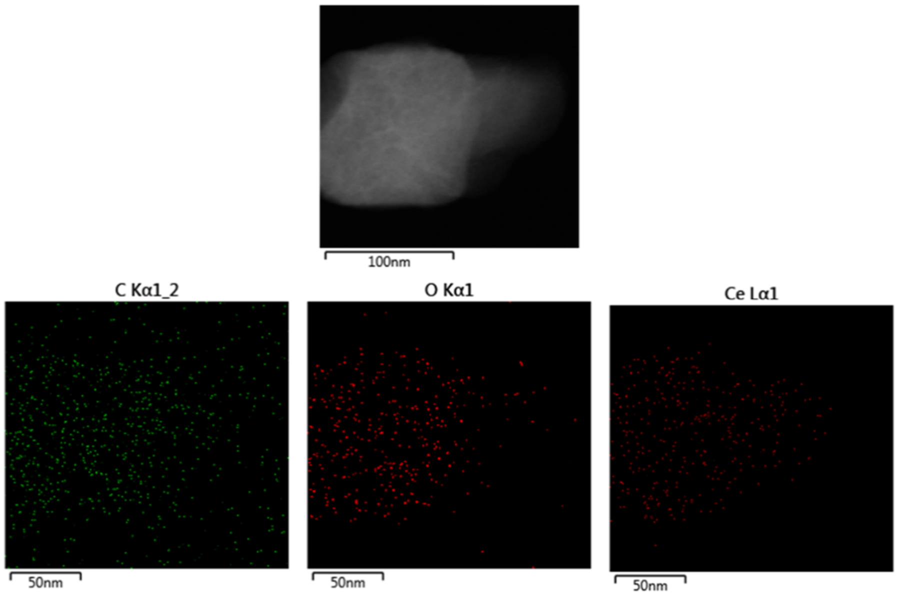

Figure S16. STEM images of the fresh UiO-66(Ce). The EDX analysis shows an average Ce composition of 31.9 wt% taking more than 5 different particles.

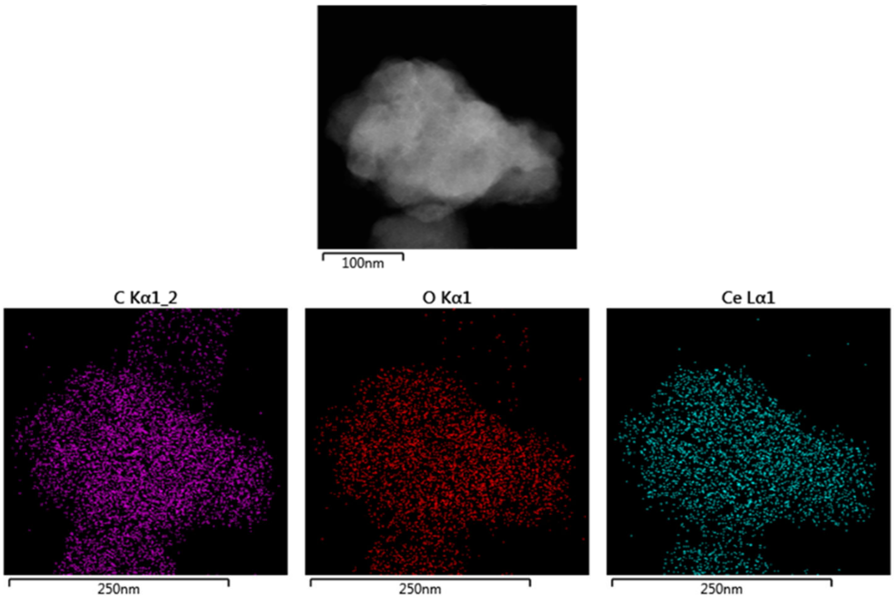

Figure S17. STEM images of the reused UiO-66(Ce). The EDX analysis shows an average Ce composition of 29.7 wt% taking more than 5 different particles.

Synthesis of additional Ce-containing MOFs for comparison purposes

Synthesis of UiO-66(Ce) with larger particle size

This synthesis has been carried out following a recipe described in the literature.[5] 39 mg of the Ce_6 cluster (see experimental section for details) was mixed with water (0.5 mL) in a 10 mL glass-vessel reactor. Separately, 1 g de benzoic acid and 33 mg of terephthalic acid were dissolved in DMF (2 mL) under sonication. Both solutions were then added to the reactor containing the Ce cluster and this reactor was transferred to a 100°C oven for 18 hours. After that, the resulting pale yellow solid was centrifuged and washed two times with DMF and finally with acetone.

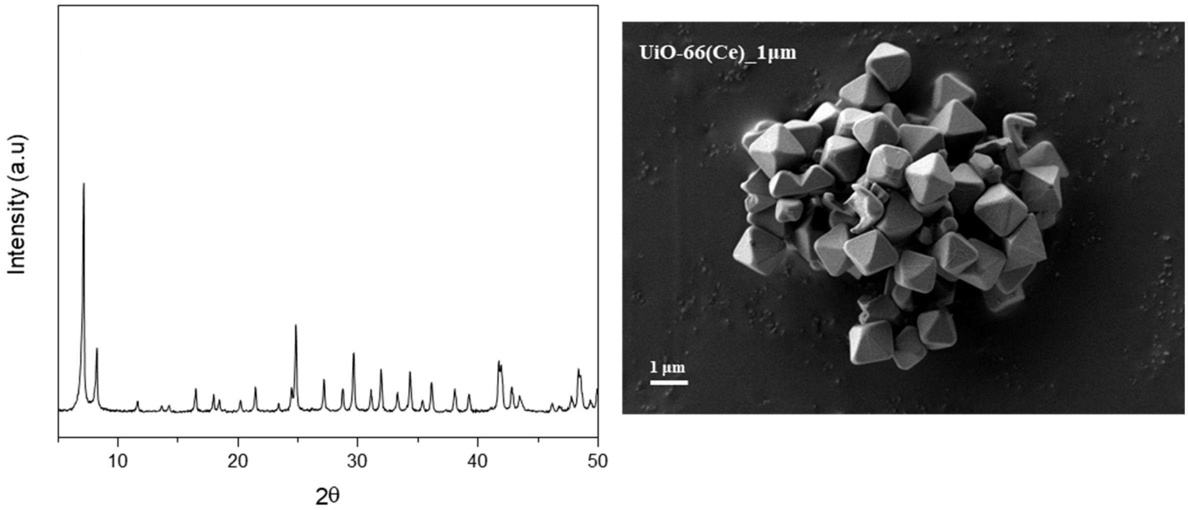

Figure S18. PXRD pattern and FE-SEM image of the UiO-66(Ce) material with larger particle size (UiO-66(Ce)_1µm).

Synthesis of Ce-MOF-808

This synthesis has been carried out following a recipe described in the literature.[6] In a 10 mL glass-vessel reactor, 22.40 mg of trimesic acid were mixed with DMF (1.20 mL) and formic acid (0.25 mL). Then, an aqueous solution of cerium(IV) ammonium nitrate (0.60 mL, 0.53 M) was added to the mixture. 4 Pyrex reactors were heated at 100°C and magnetically stirred for 15 min in a steel block. The resulting yellow solid was centrifuged and washed three times with DMF and finally with acetone.

Synthesis of Ce-MOF-801

This synthesis has been carried out following a recipe described in the literature.[7] In a 50 mL glass-vessel flask, 610 mg of cerium(IV) ammonium nitrate were mixed with water (6 mL) and formic acid (1.5 mL). Then, 130 mg of fumaric acid were added to the mixture. The solution was heated and magnetically stirred at room temperature for 12 hours. The resulting pale yellow solid was centrifuged and washed three times with DMF and finally with acetone.

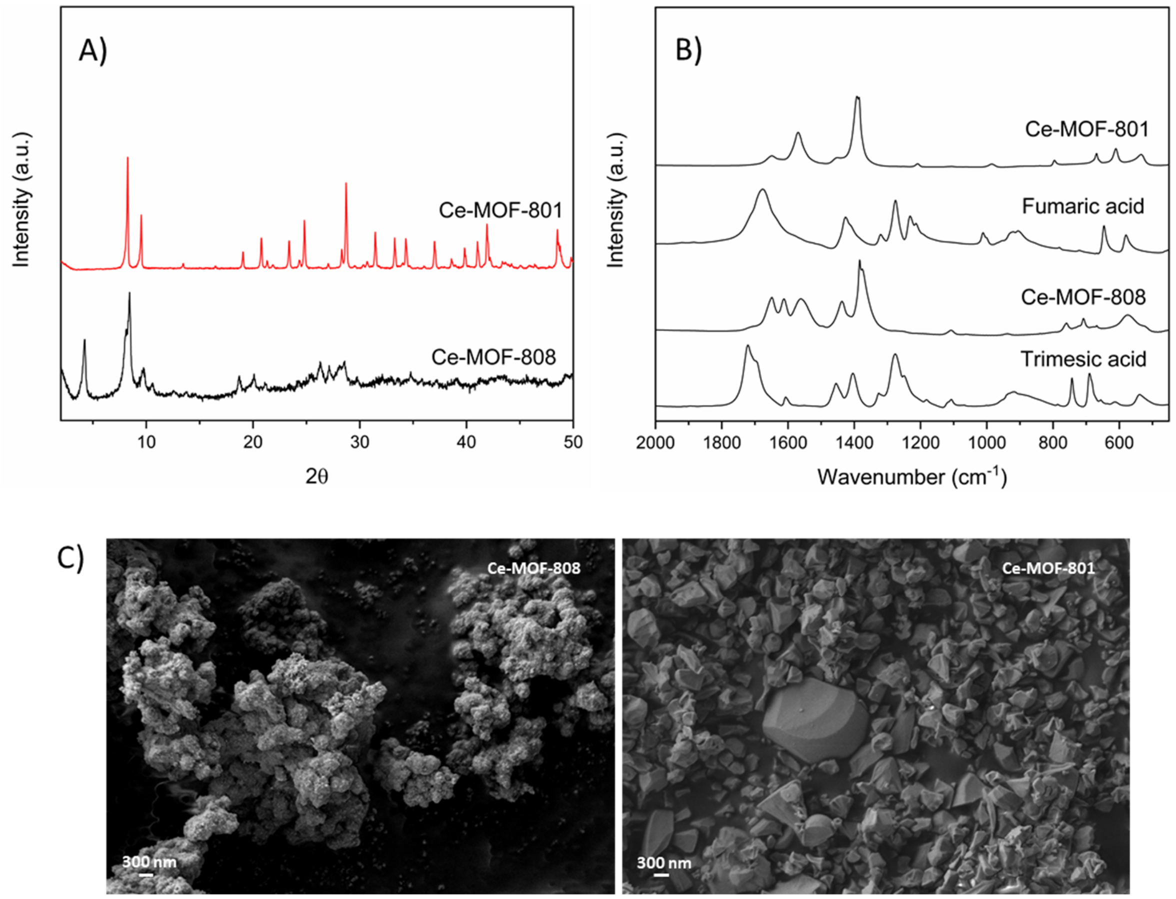

Figure S19. A) PXRD patterns, B) FTIR spectra and C) FE-SEM images of the Ce-MOF-808 and Ce-MOF-801 materials.

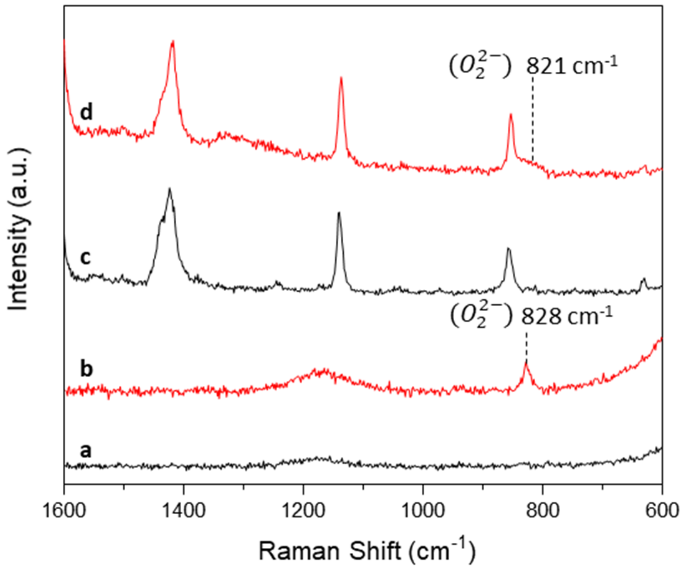

Figure S20. Raman spectra of pure nanocrystalline ceria a) and UiO-66(Ce) c) in He atmosphere (black lines) and after their exposure to an aerobic stream at 140°C b) and d), respectively.

Table S1. Binding energies of the Ce^{4+} and Ce^{3+} components in the nanoceria and UiO-66(Ce) samples.

**Sample**	**Ce^{4+} (BE;eV)**	**Ce^{3+} (BE; eV)**	**Ce^{4+}/Ce^{3+}**
nanoceria	882.5 (ν) 888.9 (ν”) 898.1 (ν”’)	880.1 ($ν^0$) 884.7(ν’)	7.4
UiO-66(Ce)	883.2 (ν) 889.5 (ν”) 898.5 (ν”’)	880.4 ($ν^0$) 885.8 (ν’)	7.9

Table S2. Calculated values for TOF (mmol product/mmol Ce. h), product yield and TMB conversion after 21 h for the oxidative halogenation reaction of TMB using UiO-66(Ce) and nanoceria as catalysts.

Sample	TOF value (mmol product/mmol Ce . h)	Product yield (%)	TMB conversion (%)
nanoceria	0.037	45	46
UiO-66(Ce)	0.121	84	98

Table S3. Re-calculated values for TOF (mmol product/mmol Ce. h) for the oxidative halogenation reaction of TMB considering only the surface sites on nanoceria and unsaturated sites on UiO-66(Ce).

**Sample**	**TOF (h^{-1})**
UiO-66(Ce)	0.87
nanoceria	0.47

Table S4. Elemental analyses of the UiO-66(Ce) material in its as-prepared form and after one-cycle oxidative halogenation reaction.

**Sample**	**C [a] (%wt)**	**H [a] (%wt)**	**N [a] (%wt)**
UiO-66(Ce) as synthesized	20.4	2.4	0.8
UiO-66(Ce) recovered after reaction	22.9	2.6	0.9

Table S5. TMB conversion, mono-brominated product selectivity and molar balance obtained for the oxidative halogenation reaction when using UiO-66(Ce) as catalyst analyzing the reaction mixture and considering the TMB adsorbed on the catalyst.

**TMB oxidative halogenation with UiO-66(Ce) after 21 hours**	**Conv. (%)**	**Selec. (%)**	**Molar Ba. (%)**
Reaction crude	98.0	85.7	86.0
Including TMB adsorbed in the catalyst	86.7	96.9	97.3

Calculation of the TMB amount adsorbed on UiO-66(Ce)

62 mg of catalyst was recovered from reaction media after one-cycle reaction. Taking into account that additional 2.5 %wt of C was detected by thermogravimetric analysis in the UiO-66(Ce) material after the oxidative halogenation reaction (see Table S4), the extra amount of C in mmol can be calculated as follows:

$$\frac{62\ mg\ solid\ x\ 2.5}{100} x \frac{1\ mmol\ C}{12\ mg\ C} = 0.13\ mmol\ C$$

Considering that 1 mmol of TMB has 9 carbon atoms, the total amount of TMB adsorbed in the catalyst can be calculated as follows:

$$0.13\ mmol\ C\ x\ \frac{1\ mmol\ TMB}{9\ mmol\ C} = 0.014\ mmol\ TMB$$

If the adsorbed TMB is added to the TMB amount detected by GC after 21 hours, the overall selectivity of towards mono-brominated TMB is enhanced from 86 to 97%.

Table S6. TMB conversion, mono-brominated product selectivity and molar balance obtained for the oxidative halogenation reaction in non-polar solvents when using UiO-66(Ce) as catalyst.

**Solvent**	**Conv. (%)**	**Selec. (%)**	**Molar Ba. (%)**
o-xylene	28.4	12.3	75.1
n-decane	63.1	12.6	44.9

Table S7. Calculated values for TOF (mmol product/mmol Ce. h), product yield and TMB conversion after 21 h for the oxidative halogenation reaction of TMB using different Ce-containing MOFs.

Sample	TOF value (mmol product/mmol Ce . h)	Product yield (%)	TMB conversion (%)
UiO-66(Ce)_1µm[b]	0.069	67	>99
Ce-MOF-808[b]	0.281	87[a]	94[a]
Ce-MOF-801[b]	0.056	78	93

[a] After 7 h.

[b] 64 mg.

References:

(1) Lammert, M.; Wharmby, M. T.; Smolders, S.; Bueken, B.; Lieb, A.; Lomachenko, K. A.; De Vos, D.; Stock, N. Cerium-Based Metal Organic Frameworks with UiO-66 Architecture: Synthesis, Properties and Redox Catalytic Activity. *Chem. Commun.* **2015**, *51* (63), 12578–12581.

(2) Villoria-del-Álamo, B.; Rojas-Buzo, S.; García-García, P.; Corma, A. Zr-MOF-808 as Catalyst for Amide Esterification. *Chem. – A Eur. J.* **2021**, *27* (14), 4588–4598.

(3) Jia, C.; Cirujano, F. G.; Bueken, B.; Claes, B.; Jonckheere, D.; Van Geem, K. M.; De Vos, D. Geminal Coordinatively Unsaturated Sites on MOF-808 for the Selective Uptake of Phenolics from a Real Bio-Oil Mixture. *ChemSusChem* **2019**, *12* (6), 1256–1266.

(4) Estes, S. L.; Antonio, M. R.; Soderholm, L. Tetravalent Ce in the Nitrate-Decorated Hexanuclear Cluster [Ce6 (M3-O) 4 (M3-OH) 4] 12+: A Structural End Point for Ceria Nanoparticles. *J. Phys. Chem. C* **2016**, *120* (10), 5810–5818.

(5) Wasson, M. C.; Otake, K.; Gong, X.; Strathman, A. R.; Islamoglu, T.; Gianneschi, N. C.; Farha, O. K. Modulation of Crystal Growth and Structure within Cerium-Based Metal–Organic Frameworks. *CrystEngComm* **2020**, *22* (47), 8182–8188.

(6) Lammert, M.; Glißmann, C.; Reinsch, H.; Stock, N. Synthesis and Characterization of New Ce(IV)-MOFs Exhibiting Various Framework Topologies. *Cryst. Growth Des.* **2017**, *17* (3), 1125–1131.

(7) Dai, S.; Nouar, F.; Zhang, S.; Tissot, A.; Serre, C. One-Step Room-Temperature Synthesis of Metal(IV) Carboxylate Metal—Organic Frameworks. *Angew. Chemie Int. Ed.* **2021**, *60* (8), 4282–4288.
